# Current Challenges of Transcription Compartmentalization Research

**DOI:** 10.1002/advs.76703

**Published:** 2026-07-23

**Authors:** Thomas Quail, Sina Wittmann

**Affiliations:** ^1^ European Molecular Biology Laboratory (EMBL) Cell Biology and Biophysics Unit Heidelberg Germany; ^2^ Institute of Molecular Biology (IMB) Mainz Germany; ^3^ Institute for Quantitative and Computational Biosciences (IQCB) Johannes Gutenberg University Mainz Germany

**Keywords:** condensation, intrinsically disordered proteins, macromolecular assembly, molecular biophysics, multivalent binding, nucleus, RNA polymerase II, single molecule imaging, superresolution, transcription (biology)

## Abstract

Transcription is spatially organized in the nucleus, concentrating transcription factors, coactivators, and RNA polymerase II into dynamic, membraneless compartments. Such assemblies have been described in many ways, from small complexes of defined stoichiometry to membraneless biomolecular condensates. We argue that these are not competing descriptions but positions along a single continuum, and propose “transcriptional compartment” as an umbrella term for it. We then turn to the functional literature and ask what these compartments have been shown to do, and what they have not. The clearest evidence for a functional role of condensation may emerge in contexts where transcription must generate switch‐like responses, such as cell‐fate decisions, stress responses, environmental sensing, and disease. Much of the remaining biology, however, sits in the small, transient assemblies between the two extremes. These are the hardest to study, in part because they fall below the diffraction limit of light microscopy and because the interactions that drive their formation usually also carry out their function, so the two cannot be perturbed independently. We survey the experimental and computational toolkit available for this problem and outline the open methodological challenges that remain.

## Graphical abstract

Transcription factors, coactivators, and RNA polymerase II assemble into transcription compartments ranging from small, defined complexes to liquid‐like condensates. This review unifies these seemingly competing descriptions along a single continuum and asks what these compartments have been shown to do, and what they have not, revealing that the most elusive, transient assemblies are also the hardest to pin down mechanistically and probe experimentally. It maps the experimental and computational toolkit available and outlines the methodological challenges still standing in the way.

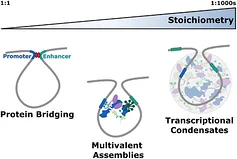

## 1 Introduction

Over the past decades, functional, genetic, and bioinformatics‐based studies have mapped the sequence‐specific recognition motifs (typically 6–12 base pairs) of most human transcription factors (TFs) [1]. Accurately predicting TF binding localization across the genome, however, remains a major challenge despite recent progress using deep learning tools [2]. This is perhaps unsurprising, as accumulating evidence has revealed that gene expression operates at length scales beyond individual motif recognition [3]. Cis‐regulatory elements such as enhancers and promoters typically span hundreds of base pairs and often contain clusters of TF motifs [4], suggesting that cooperative interactions among cofactors play a key regulatory role. An additional problem is that enhancers and promoters can be separated by up to 100s of kbps in the linear genome, and, for proper transcriptional control, these enhancers and promoters must be brought into close proximity [3]. How have cells evolved mechanisms to overcome these length scale problems for such essential biological functions?  In this review, our central focus is on transcription compartmentalization at the length scale of enhancer‐promoter contacts and how TFs, cofactors, chromatin regulatory factors, and RNA polymerases act to establish environments that are conducive to gene expression. Owing to its importance, there has been much essential work on how transcription is compartmentalized at this length scale, which we will briefly review for context.

## 2 General Models of Enhancer‐Promoter Contacts

Several distinct models have been proposed for how enhancers and promoters communicate, ranging from direct protein bridges to chromatin loop extrusion and condensate‐mediated contact, each making different predictions and are distinguishable by different experimental signatures. The most prominent models are described in detail below, while a comprehensive overview is provided in Table 1.

**TABLE 1:** Overview of models for enhancer‐promoter communication and their associated assembly types.

Model	Definition	Stoichiometry	Discriminating prediction	Experimental feasibility	Key limitations	References
**Bridging**	A protein bridge holds enhancers (E) and promoters (P) in close proximity by binding determinants at both elements.	Defined: a structured protein link spans the two elements.	Disrupting the **bridge specifically** (not the participating factors' other functions) abolishes the E‐P loop while the factors remain chromatin‐bound.	**Challenging but feasible** — separation‐of‐function mutants.	Distinguishing the bridge from what it recruits can be hard.	—
Direct TF‐TF bridge	A single sequence‐specific TF binds its motifs at **both** E and P and self‐associates, linking them directly.	—	—	**Feasible/shown** — YY1 dimerization mutants.	Bridge‐interface mutants hard to design cleanly.	[7]
Indirect bridge via cofactor	A **non‐DNA‐binding** cofactor is recruited through partner TFs at both elements and self‐associates to bridge them.	—	—	**Feasible/shown** — LDB1 dimerization‐domain rescue uncouples looping from transcription. **Feasible/refuted** — Mediator was shown to be dispensable for tethering DNA	In vivo geometry hard to resolve; overlaps with cluster regime.	[10]
**Loop extrusion**	Cohesin extrudes DNA, stalled at CTCF/TF/Mediator/Pol II barriers, juxtaposing E and P within a TAD.	Defined extrusion motor; variable loop content.	Loops are **CTCF‐motif‐orientation‐dependent** and grow/shrink processively; abolished by cohesin/WAPL/NIPBL perturbation but **insensitive** to condensate‐dissolving agents (e.g. 1,6‐hexanediol).	**Feasible** — motif inversion; single‐molecule + Micro‐C; hexanediol controls.	Acute depletion often yields only mild transcriptional effects; importance for E–P contact formation unclear.	[182, 183, 184]
**Transcription factories**	Pol II‐rich foci transcribing multiple templates; classically, polymerase is **fixed** and DNA is reeled through.	Variable: multi‐gene, Pol II‐rich.	Polymerase is **spatially immobile/long‐lived** and the template moves through it.	**Feasible/refuted** — live single‐molecule imaging shows Pol II clusters are transient (∼5 s; ∼8 s), dynamic, and not statically assembled, contradicting the immobile‐factory prediction.	Strong static model not supported; superseded by dynamic cluster/condensate views; largely historical.	[21, 29, 69]
**Contact via multivalent assembly**	E and P co‐localize because both are captured in / wetted by the **same** body.	Variable: no fixed stoichiometry.	Several elements share **one** body **simultaneously**, as supposed to fast sequential pairwise E–P loop contacts.	**Feasible but ambiguous** — multi‐element co‐residence imageable; separating simultaneous co‐residence from rapid serial looping is the hard part.	Co‐localisation alone does not prove a phase; resolution‐limited.	—
Sub‐saturation clusters / hubs	Transient, chromatin‐nucleated clusters forming **below** c_sat_; no macroscopic phase required.	Variable: sub‐saturation.	The **same** factor activates transcription **below** its critical concentration (no droplet), as well as above it — activation tracks multivalent interaction, **not** the phase transition.	**Shown** — optogenetic constructs comparing matched TF ± droplet show activation enhanced by multivalency independent of phase separation, with direct binding‐kinetics readout.	Hard to distinguish from a small condensate at the diffraction limit.	[63, 185]
Surface condensation	Regulatory chromatin provides a wetting surface (prewetting), so clusters condense at concentrations below bulk c_sat_.	Variable: surface‐coupled.	Condensation occurs **only on the chromatin surface** below bulk c_sat_; clusters are **non‐spherical** and show **no fusion or Ostwald ripening** — unlike spherical bulk droplets.	**Demanding/shown** — super‐resolution in zebrafish shows variable shapes, no ripening; clusters transiently connect/separate.	Boundary with the other two condensate sub‐rows genuinely fuzzy in vivo.	[42, 43, 47]
LLPS condensate	Weak multivalent IDR–IDR interactions drive an **equilibrium** liquid–liquid phase transition **above** c_sat_.	Variable: above‐saturation.	Assemblies are **spherical**, **fuse**, and show **Ostwald ripening** + concentration buffering — the full equilibrium‐LLPS signature, absent in the two sub‐rows above.	**Demanding** — droplet hallmarks measurable in vitro; in‐cell c_sat_ thresholds and coarsening at/below resolution.	Bulk equilibrium LLPS not established in vivo and superseded for many loci by sub‐saturation / surface‐condensation accounts.	[50]
**TAG (TF activity gradient)**	*Contact‐independent*. Enhancer‐localized enzymatic activity (e.g. p300/CBP acetylation) creates a diffusible gradient of activated TFs reaching nearby promoters; HDACs limit it. **Agnostic to what assembles at the enhancer — condensate or defined complex**.	N/A: not an assembly; diffusible gradient.	Productive transcription occurs **without stable E–P contact** (proximity but no contact) and is broken by **HAT/HDAC perturbation** rather than by contact/looping disruption — the only model predicting activity decoupled from physical contact.	**Emerging** — two‐color distance‐vs‐output imaging can show activity without contact; the diffusible activated‐TF gradient itself remains hard to visualize directly.	Apparent ‘no contact’ may reflect incomplete labelling (a bridge via an unimaged species would be missed); direct evidence for the gradient still limited.	[186]

Abbreviation: Csat, saturation concentration.

### 2.1 Bridging

Direct molecular bridging by TFs at regulatory DNA elements represents one of the early models thought to drive enhancer‐promoter contacts. YY1 and GAGA are among the most well‐studied TFs thought to drive bridging in both *Drosophila* and mammalian contexts [5, 6]. YY1 binds to specific DNA motifs at both enhancers and promoters, and its ability to dimerize enables it to physically link these two distant regulatory elements [7]. Loss of YY1 binding sites or depletion of YY1 protein disrupts enhancer‐promoter contacts and reduces target gene expression, particularly at developmentally regulated loci [7]. Similarly, GAGA binds to GA‐rich sequences and has been shown to mediate long‐range interactions, where it can facilitate enhancer‐promoter contacts independent of CTCF or cohesin [6].

But there are other ways to build a molecular bridge. Multivalent protein complexes can also anchor via protein‐protein interactions with DNA‐bound TFs at both enhancers and promoters. For example, the protein Ldb1 and its *Drosophila* homologue Chip interact with LIM‐domain TFs, which triggers dimerization and the formation of higher‐order oligomers, thereby bridging different DNA regions bound by TFs [8]. In addition, the Mediator complex was proposed to bridge DNA in specific contexts by binding to an enhancer‐bound TF through its tail domain, while interacting with the pre‐initiation complex at the promoter via its head and middle modules [9]. However, rapid depletion of Mediator leaves most enhancer‐promoter contacts intact, suggesting that it acts as a functional rather than an architectural bridge [10]. Importantly, these bridging interactions are thought to consist of stereotypic protein:protein interactions involving folded domains that form stable bridges over long time scales [11]. Different models have been proposed over the years suggesting variants of the bridging model described here involving additional layers of regulation beyond oligomerization and are reviewed elsewhere [5, 11, 12].

### 2.2 Looping

Chromatin enzymes are also thought to drive molecular mechanisms that can then bridge distal DNA regions. The most prominent example is that of chromatin looping, a mechanism that is driven by the ring‐shaped protein complex cohesin, which is loaded onto the DNA at nucleosome‐free regions. After loading, cohesin pulls DNA through its ring‐like structure in a symmetric manner (from both sides) [13, 14, 15] through a process termed loop extrusion. Interactions of cohesin with the DNA‐binding protein CTCF bound to its sequence‐specific motif are thought to stall loop extrusion by cohesin, thus stabilizing the loop, and thereby creating a topologically associated domain (TAD) [16]. In addition, chromatin loops that are not anchored to genomically‐encoded CTCF sites were shown to drive tissue‐specific enhancer‐promoter contacts within a TAD [17, 18]. At these sites, bound TFs, Mediator, and transcribing RNA polymerase II (Pol II) appear to act as alternative barriers or stalling points for cohesin extrusion, rather than CTCF [19, 20]. However, perturbations often result only in mild effects on transcription [12].

### 2.3 Biomolecular Condensates

Cell biological imaging studies from Peter Cook's group in the 1990s as well as other groups revealed that transcriptional regulators and factors clustered together in space [21]. Based on light and electron microscopic evidence as well as mass spectrometry, they proposed that Pol II and other transcriptional proteins form stable transcription ‘factories’ to which DNA is translocated for transcription [21]. Since then, various terms have been used to describe these transcriptional sites including hubs [22], bodies [23], clusters [24], foci [25], compartments [26], and most recently, condensates [27, 28], which reflects the evolving understanding of their nature. It was not until 2013 that a paper by Cissé et al. not only confirmed the existence of Pol II clusters but also challenged the view of stable, long‐lived structures [29]. Further live cell imaging experiments followed that revealed that promoter‐enhancer contacts can exhibit short lifetimes on the order of seconds or even less [27, 28, 30, 31]. This fundamentally changed our understanding of how the transcriptional machinery organises inside cells and  led to a new model, the transcriptional condensate model, in which weak, multivalent interactions mediated by intrinsically disordered regions (IDRs) of transcriptional regulators drive the formation of biomolecular condensates [23]. These condensates are thought to be particularly enriched at loci termed super‐enhancers, where they facilitate the concentration of TFs, coactivators such as the Mediator complex and Pol II, thereby promoting the spatial clustering of regulatory DNA elements [27, 28, 32, 33, 34].

Although super‐enhancer condensates have received most attention, similar assemblies have also been observed at housekeeping genes, which do not engage with cell‐type‐specific enhancers and instead rely on constitutive expression to sustain general cell metabolism and growth [35]. Housekeeping genes tend to cluster along the chromosome [36, 37], which enables their promoters to physically interact, allowing groups of housekeeping genes to be co‐organized within shared assemblies [38]. Consistent with this, the activity of each housekeeping gene depends on the expression of its neighbors [38], which suggests that promoter condensates may co‐regulate essential cellular pathways. Similar to other enhancer‐promoter clusters, these promoter‐promoter clusters can concentrate other transcriptional proteins such as Pol II and the Mediator complex, but their formation is driven by a protein called Ronin [35].

## 3 Biophysical Principles of Transcriptional Condensates

Interpreting these assemblies mechanistically requires dissecting their biophysical principles, with biomolecular condensation emerging as one prominent, albeit debated, model. A framework often invoked to describe them is liquid‐liquid phase separation (LLPS) [39]. Thermodynamically, LLPS balances the energetic gain from favourable intermolecular interactions against the entropic cost of forming a well‐defined surface demarcating the dilute and condensed phases [39, 40]. This balance defines a saturation concentration (c_sat_) above which the system demixes through an abrupt, first‐order transition. Thus, modest changes in local TF concentration, valency, or interaction strength can flip the system between one regime and the other, giving condensate formation a switch‐like rather than gradual character [40]. Crossing c_sat_ is nonetheless a stochastic event, as nucleation theory holds that small assemblies form and dissolve continually, with only those exceeding a critical size overcoming the energetic cost of creating a surface [40, 41]. In the nucleus, this barrier can be lowered by heterogeneous nucleation on chromatin, where DNA, histones, and sequence‐specific motifs provide binding sites that locally raise effective concentration and valency [42]. Enhancers and super‐enhancers, dense in TF motifs, could thus act as spatially fixed nucleation platforms, though this remains experimentally undemonstrated in cells.

**FIGURE 1:**
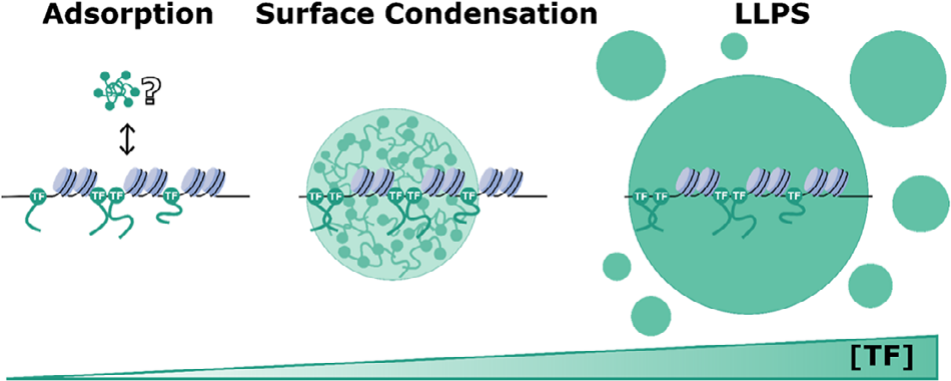
Biophysical model of surface condensation on DNA. Schematic illustrating how TF concentration determines the mode of assembly on DNA. Whether TFs in general are monomeric or form small clusters in solution and which form is binding to DNA remains unknown. At low concentrations, TFs adsorb to DNA, locally increasing their effective concentration. Proximal molecules are able to form IDR‐mediated interactions, leading to the formation of surface condensates that remain restricted to the DNA at intermediate TF concentrations. Above c_sat_, LLPS occurs in the bulk solution, and condensates are no longer confined to the DNA surface.

More specifically, increasing attention has focused on surface condensates driven by capillary‐like forces, which form at interfaces such as chromatin fibers, nuclear membranes, or pre‐existing nuclear bodies [42, 43, 44]. Two regimes need to be distinguished here. At low concentrations, individual molecules adsorb to the surface, forming a thin adsorbed layer in which they can still engage in multivalent contacts with one another. Through prewetting, a surface with affinity for a molecule can then stabilize a much denser condensed layer at concentrations below the bulk saturation concentration, where no bulk phase would otherwise form (Figure 1) [45, 46]. Approaches using single molecule biophysics have shown that in vitro TFs can form surface condensates that pull on DNA [43, 47] and that can bind in a sequence‐specific manner [42]. Surface condensates are selective, force‐generating nuclear bodies that can up‐concentrate transcriptional machinery at physiological concentrations. These condensates can both co‐condense DNA but can also generate stresses that deform the chromatinized network [48]. A recent study from the Brangwynne lab used optogenetic approaches to demonstrate that it was possible to reposition genomic loci using photo‐induced biomolecular condensates through these capillary‐like forces [49].

These physical distinctions motivate how we use the associated terms. A *complex* is an assembly of defined, low‐copy‐number stoichiometry held together by structured interfaces, as in the pre‐initiation complexes of RNA polymerases or a TF bridge. A *condensate* is the general term for components locally concentrated into a distinct high‐density phase, covering both bulk phase separation, the transition above c_sat_ that yields freely suspended droplets, and the surface or prewetting condensates that form on chromatin below it. A *cluster* or *hub* is an assembly in which factors are only seen to concentrate, without the density transition itself having been tested. Because it is often unclear whether these terms mark genuinely distinct physical states or simply different words for the same thing, we propose *transcriptional compartment* as an umbrella term spanning the whole range, from complex through cluster to condensate, grouped by their shared role of concentrating the transcriptional machinery at regulatory DNA (Table 1).

It is worth noting that the original proposal of LLPS in the context of transcription organization by Hnisz et al. [50] was a conceptual framework rather than a direct demonstration. Although it has since been cited extensively, this reflects the influence of the idea more than settled agreement, and the evidence gathered since speaks less to bulk LLPS than to condensation in the broader sense. Nonetheless, it sparked a huge wave of interest in biomolecular condensation as an organizing principle for transcription.

## 4 Biological Functions of Transcriptional Compartments

It is well accepted that, in specific contexts, transcription takes place in discrete foci and that the weak, transient interactions of TFs, cofactors, and Pol II are essential for proper regulatory control. What the condensate model added to this long‐standing picture was a set of attractive hypotheses, including abrupt formation/dissolution mechanisms, selectivity, up‐concentration of factors, amongst others. But whether these biophysical predictions translate into genuine biological function remains an open question. Therefore, we wanted to know what the evidence actually tells us about the biological functions of transcriptional condensates. Throughout this section, we use *condensate* when the cited study provides evidence that the assembly is phase‐separated, and other terms when the assembly has been observed but its phase‐separated nature has not been established.

### 4.1 Up‐Concentrating the Transcriptional Machinery Boosts Gene Expression

A central hypothesis of transcriptional condensate function is that they locally concentrate TFs, coactivators, and Pol II at enhancers and promoters, thereby driving expression of target genes. The mechanistic rationale is mass action: enriching enzymes and substrates in a small volume increases the frequency of productive encounters and accelerates the reaction [51]. For transcription, this would translate into a higher rate of pre‐initiation complex assembly and Pol II initiation, and therefore into higher mRNA output [52]. Condensates would accordingly be expected to matter mostly for genes that need to be expressed at high levels, rather than for genes whose moderate to low expression is already supported by standard recruitment.

This principle has been validated in non‐transcriptional condensates in vitro. For example, in an engineered SUMOylation system, recruitment of the enzyme cascade into protein condensates accelerated SUMO conjugation by up to 36‐fold as compared to the dilute phase [53]. Similar effects have also been demonstrated for RAS activation and for lipase condensates [54, 55]. Therefore, condensates can, in principle, accelerate enzymatic reactions through the predicted mechanism. However, all of these measurements have been performed in vitro using purified or reconstituted systems. For Pol II transcription, in vitro reconstitution has not been achieved, and full purification of the active transcriptional machinery remains technically very challenging, further limiting quantitative tests of the proposed mass‐action mechanism.

Despite this gap, transcriptional condensates have been correlated with biological contexts requiring high gene expression, including development, stress response, or in disease states characterized by aberrant gene expression [56]. For example, super‐enhancer condensates enriched in the ubiquitous transcriptional coactivators BRD4 and Mediator complex were shown to ensure the expression of cell‐identity genes to maintain the pluripotent state of mouse embryonic stem cells [27, 28] and large condensates enriched in the TFs Nanog and Sox19b as well as Pol II and Cdk9 control zygotic genome activation in zebrafish [23]. Condensate formation has further been associated with cell‐fate decisions, such as cardiovascular lineage specification [57] and adipocyte differentiation [58]. Transcriptional condensates have also been reported in rapid stress responses, most notably driven by heat shock factor 1 (HSF1) [59] and at super‐enhancers driving aberrant oncogene expression in cancer [60].

There is also evidence that condensates concentrate components of the transcriptional machinery and that this may enhance transcription. In cells, light‐induced condensation of synthetic IDR‐containing TFs can increase transcriptional output by up to ~5 fold [61], and optogenetically nucleated TAF15 condensates amplify transcription by ∼2‐fold [62]. However, there is also evidence in the opposite direction. Trojanowski et al. compared TFs with and without phase‐separation capacity using a reporter‐based array and concluded that phase separation per se is not required for transcriptional activation [63]. Rather, activation strength scaled with the multivalent interaction propensity of the activation domain, while the appearance of liquid‐like droplets at higher expression levels had a neutral or inhibitory effect [63].

This inhibitory effect is itself informative, and connects to a much older observation. “Squelching” experiments showed that high concentrations of activation domains can paradoxically inhibit transcription by titrating limiting components of the transcriptional machinery away from their target genes [64]. Modern condensate experiments revisit this regime. In vitro, MED1‐IDR added to a transcriptionally‐competent nuclear extract sequesters BRD4 and Pol II into droplets and represses transcription in a dose‐dependent manner directly demonstrating that condensate formation can reduce transcriptional output [27]. In cells, single‐molecule tracking experiments showed that the same FUS‐IDR‐fused p53 construct enhances target gene expression at low expression levels but at higher expression forms condensates that detach p53 from DNA‐dense regions and downregulate target gene transcription [65]. In principle, to convincingly demonstrate that condensation enhances transcription in cells, expression levels must therefore be tuned within a narrow window: high enough to nucleate a condensate at the chromatin‐bound target site, but not so high that off‐chromatin condensation sequesters components away from the gene. Taken together, the available evidence supports a role for multivalent IDR interactions—and perhaps condensate formation—in driving high transcriptional output, with the precise contribution probably depending on the specific TF, locus, and cellular context.

### 4.2 Transcriptional Bursting

A second proposed function of transcriptional condensates is to drive transcriptional bursting, which is the stochastic, episodic pattern in which genes are transcribed in short pulses of activity separated by longer inactive periods, with on‐ and off‐times typically in the range of minutes to hours [66]. A central question is how fast‐time scale molecular events (~<seconds), such as TF‐DNA binding, are translated into the much slower bursting kinetics (∼minutes, ∼hours). The condensate framework offers a physically appealing answer: when a condensate forms at a locus, it concentrates Pol II and coactivators and enables a burst of mRNA production; when it dissolves, transcription ceases [50]. Two physical features of condensation make this attractive as a mechanism for bursting. First, condensate nucleation is a dynamic process that integrates and encodes dynamic information across a range of time scales at least in vitro [67]. Second, condensate formation requires crossing a nucleation barrier, which is a stochastic event that, once it occurs, drives rapid assembly of the condensate. The barrier therefore produces an all‐or‐nothing switch between two states: long quiet periods with no transcription punctuated by sudden condensate formation and a burst of mRNA production [62].

Several endogenous systems provide cellular evidence consistent with cluster dynamics matching the timing of bursts. In early *Drosophila* embryos, live imaging of endogenously tagged proteins revealed an ordered cascade of factor clustering at the appropriate time scale to drive a burst, with nascent transcription itself dispersing the clusters to produce a self‐limiting burst [68]. Similarly, at the β‐actin locus in mouse embryonic fibroblasts, Pol II cluster lifetime increased roughly 3 fold upon serum stimulation, which was linearly proportional to the number of nascent transcripts subsequently produced [69]. Similar correlations between cluster dynamics and bursting have since been reported in *Drosophila* and human breast cancer cells [70, 71]. The same logic appears to extend beyond single loci: in *Drosophila* embryos, paralogous genes separated by long genomic distances show coupled bursting that depends on promoter‐proximal tethering elements, consistent with shared TF clusters coordinating bursts at distant genes [72].

However, more recent work complicates this picture. At the *Sox2* locus in mouse embryonic stem cells, bursting was modulated not by the formation of condensates, but by the proximity of pre‐existing Pol II/Mediator condensates. Bursts were enhanced when the condensate transiently approached the gene to within <1 µm, which led the authors to propose a “three‐way kissing” model of contact between the condensate, the gene, and downstream regulatory elements [31]. A different kind of dissociation has been observed in the Drosophila Notch system, where Mastermind clusters form in essentially all signaling‐competent cells, but only about a third of these cells produce mRNA in any given window, suggesting that clusters are necessary but not sufficient for bursting [70]. The most direct functional challenge comes from budding yeast. Although Gal4 forms clusters that overlap with the *GAL* loci, mutations that perturb cluster formation do not affect target gene expression. Instead, clusters facilitate target search (as discussed further in Section 4.6) but do not drive bursting [73]. In a follow‐up study, simultaneous tracking of Gal4 binding and *GAL10* transcription pointed to cooperative binding and rapid exchange of multiple Gal4 molecules across the four promoter binding sites as the mechanism sustaining bursts [74].

Taken together, direct evidence that condensate or cluster formation nucleates and/or dissolves a bursting event has not been presented. In yeast, the link is clearly absent. In metazoans, condensates and bursts are correlated at multiple endogenous loci, but the available evidence is not sufficient to establish causation. Moreover, clusters can form without producing a burst, and, at the Sox2 locus, condensates modulate bursting through proximity, suggesting that even where condensates and bursts coincide, the relationship may not be causal. A plausible alternative is that clustering and bursting are co‐regulated without one driving the other, either because they share an upstream cause, or because clusters tune burst properties by raising the local TF on‐rate. The relative contribution of these mechanisms is likely to depend on the locus, the cell type, and the developmental context.

### 4.3 Repression of Gene Expression

Condensate‐mediated repression spans a wide range of scales, from chromosome‐scale silencing such as X inactivation to repressive chromatin compartments like heterochromatin. Whether and how phase separation contributes to repression at these longer length scales remains debated particularly because polymer‐bridging and globule‐collapse mechanisms can account for many of the same observations, as reviewed in detail elsewhere [75, 76]. Here, we restrict the discussion to condensate‐mediated repression that act at enhancers and promoters at individual genes. Two mechanisms have been proposed: a repressive condensate may recruit and concentrate factors that repress gene expression such as heterochromatin‐promoting enzymes, or that these condensates physicochemically exclude the general transcription machinery [77].

One mode of gene‐specific repression involves the negative elongation factor (NELF), which stabilizes promoter‐proximal Pol II pausing at gene promoters [78]. Under normal conditions NELF is diffusely distributed in the nucleus, but upon stress it rapidly forms nuclear condensates. Clustering is driven by the IDR of a NELF subunit (NELFA) and is triggered by two stress‐induced modifications: dephosphorylation and SUMOylation. Condensation enhances NELF recruitment to housekeeping gene promoters and is required for their downregulation, ensuring cell viability under stress [79]. Chaperone activity by HSPA1A and DNAJB1 subsequently disperses the condensates, allowing transcription to recover once the stress subsides [80]. NELF condensation is thus a stress‐induced, reversible switch that reinforces gene repression.

A conceptually related but developmentally programmed mechanism operates during terminal erythropoiesis, where most genes are progressively silenced before the nucleus is finally expelled. During this process most Mediator subunits, including MED1, are progressively lost, while MED26 remains abundant and gradually becomes dominant. The IDR of MED26 has a stronger condensate‐forming propensity than MED1, and MED26 preferentially recruits pause‐inducing factors such as NELF, DSIF (SPT5), and PAF1, rather than the elongation machinery favoured by MED1. The composition of Mediator condensates therefore gradually shifts toward a MED26‐enriched, pausing‐prone form, which is required for the genome‐wide Pol II pausing that drives erythroid silencing [81]. In contrast to the acute, reversible NELF response, this is a slow, irreversible compositional change tied to a developmental program.

Dedicated transcriptional repressors can themselves form condensates. In embryos of the tunicate *Ciona*, the repressor Hes recruits the corepressor Groucho/TLE into viscous liquid condensates at the genes it silences [77]. A second repressor, ERF, forms analogous Groucho‐containing condensates that are dissolved upon ERK signalling, probably by ERF phosphorylation, derepressing its targets [82]. Similarly, in *C. elegans* the TF NHR‐67 forms condensates with Groucho homologues that suppress the invasive cell fate [83].  This extends the principle to dedicated repressors and shows that such repression can be switched off by an upstream signal. Although Groucho corepressors and their repressor partners are conserved in other animals including humans, whether similar condensate‐based mechanisms operate in mammalian cells has not been tested.

These examples point to condensation offering clearer advantages for repression than for activation since efficient repression requires that basal gene expression is brought close to zero, a sharp threshold that condensate assemblies are well suited to produce. In several cases, the repressive condensate also appears to physically exclude activators and the transcriptional machinery from the silenced locus, as proposed for Hes/Gro condensates [77]. A related principle has been described when repressive condensates mature into a gel‐like state in which diffusion of factors is impeded and transcription is thereby attenuated, as reported for estrogen‐receptor‐bound enhancer ribonucleoprotein complexes under chronic stimulation [32]. Repression by condensate‐mediated sequestration is also rapidly and reversibly tunable, as illustrated by the ERK‐induced dissolution of ERF condensates [82]. Whether the condensates themselves contribute beyond the underlying multivalent interactions has not been directly tested for any of these examples, but the correlation of repressive functions and condensates in diverse biological systems is worth noting.

### 4.4 Functional Switches

Beyond promoting transcription in one direction, there are examples in the literature in which condensates can act as switches, with the condensate state determining which of two distinct transcriptional outcomes is produced at the same locus. The MED1‐to‐MED26 shift during terminal erythropoiesis (as already discussed in Section 4.3) is one such case: the same compartment is rerouted from supporting elongation to pausing through a change in subunit composition [81]. In several other examples, described below, condensation itself acts as the sensor for an environmental cue such as temperature or nutrient availability, with the cue directly shifting the condensate state and thereby the transcriptional outcome.

Environmental sensing is particularly important in plants, which are exposed to vastly changing environments without the ability to adjust their internal environment in the same way animals can. In this context, condensation has emerged as a sensing modality [84]. One striking example comes from temperature sensing in *Arabidopsis thaliana* to decide when to flower, where the intrinsic temperature dependence of phase separation is itself used as the sensing mechanism. Plants need to flower at the right time of year to ensure that flowers survive and seeds can mature, and *Arabidopsis* achieves this through Early Flowering 3 (ELF3) and Frigida (FRI), which condense in opposite directions: ELF3 upon warming, FRI upon cooling. Notably, and in contrast to the activating condensates discussed in previous sections, here condensation occurs *away* from the target promoter, and the chromatin‐bound factor is the active form. ELF3 acts as a warmth sensor. In cool conditions it binds and represses growth genes such as *PIF4*, but at elevated temperature its polyglutamine prion‐like domain drives lower critical solution temperature (LCST)‐type condensation away from chromatin, derepressing these targets and permitting flowering [84, 85]. FRI acts as a cold sensor: in warm conditions it occupies the *FLC* promoter and activates this floral inhibitor, blocking flowering; prolonged cold induces FRI condensation away from *FLC*, so *FLC* is no longer activated, and flowering is licensed to proceed [86]. Although both systems ultimately enable flowering, they encode opposite cues, and *Arabidopsis* commits to flowering only when both signals have been registered.

A different mode of switching is exemplified in mammalian cells by the TF TEAD4, which can be flipped between activating and repressing the same target genes by exchanging its cofactor. TEAD4 is the downstream TF of the Hippo pathway and controls proliferation‐ and survival‐associated genes, which the cell can either drive or shut down depending on conditions. Under growth‐permissive conditions, TEAD4 binds the coactivator YAP and nucleates activating condensates at these genes [87, 88]. Under glucose starvation, TEAD4 binds the corepressor VGLL4 instead, which drives it into large repressive condensates that aggregate DNA and chromatin, contributing to stress‐induced cell death. The two condensate types are biophysically immiscible, so the cofactor that wins out determines the outcome decisively rather than producing a graded mixture. Interestingly, a short peptide derived from VGLL4 is sufficient to force TEAD4 into the repressive form, and suppresses tumour growth in mouse models, providing a separation‐of‐function manipulation that links condensate composition to transcriptional outcome [88].

In summary, the examples above describe two switching mechanisms: in one, condensation removes a chromatin‐bound factor from its target promoter; in the other, it recruits the same factor into compositionally distinct assemblies of opposite character. The two share an attractive feature for switching: the cooperative, all‐or‐nothing character of condensate assembly converts a graded input—a few degrees of temperature change for ELF3 and FRI, a shift in cofactor abundance for TEAD4—into a decisive transcriptional decision that binding equilibria alone would render as graded mixtures. For the redirection mode, the immiscibility of the two condensate types additionally enforces a binary commitment between outputs. As for the previous sections, the contribution of phase separation itself has not been directly tested, since the underlying multivalent and cofactor interactions cannot easily be separated from condensation; nonetheless, condensation provides a physical basis for the kind of sharp, decisive transitions on which many cellular decisions ultimately depend.

### 4.5 Compositional Selectivity of Transcriptional Condensates

A defining feature of nuclear condensates is that they are compositionally distinct: a transcriptional condensate, a splicing speckle, a paraspeckle, and the nucleolus coexist in the same nucleoplasm yet each concentrates its own set of proteins and nucleic acids while excluding most others, and can even sustain an internal pH distinct from the surrounding nucleoplasm [89, 90, 91]. This is notable because the interactions that build these compartments—weak, multivalent, and largely mediated by IDRs—look individually promiscuous, so how they specify composition is not at all obvious [92]. The proposed resolution is that selectivity is encoded in the sequences of the participating IDRs: each compartment is thought to be held together by a particular “molecular grammar” which is a characteristic distribution of charged, aromatic, and aliphatic residues that defines a distinct physicochemical environment [93]. A molecule is enriched if its interaction chemistry matches that environment and excluded if it does not, so that a transcriptional condensate admits the transcriptional machinery while the grammar of a splicing condensate admits splicing factors, even though both are built from similar kinds of weak IDR contacts.

The most direct demonstration that this grammar operates between distinct nuclear compartments comes from the C‐terminal domain (CTD) of Pol II, which is sorted between transcriptional and splicing condensates depending on its phosphorylation state. The hypophosphorylated CTD partitions into Mediator/MED1 condensates during initiation, whereas phosphorylation by CDK7 and CDK9 lowers its affinity for these condensates and raises its affinity for SRSF2 splicing condensates in vitro and in cells, so that a single modification rewrites which grammar the molecule matches [94]. How sequence encodes this partitioning has been dissected most cleanly for the MED1‐IDR. Work from the Sabari lab showed using cells, lysates, and in vitro droplets that alternating blocks of charged amino acids in the IDR are necessary and sufficient for selective partitioning: MED1‐IDR condensates take up Pol II together with its positive regulators while excluding negative regulators, disrupting the charge pattern abolishes this selectivity, and grafting the pattern onto unrelated proteins is enough to confer it [95]. Because the precise charge grammar also modulates which factors are enriched and the resulting gene expression output, these experiments tie a defined sequence feature directly to both condensate composition and function and is not merely a consequence of generic stickiness, but a partitioning code written into the IDR.

For the cell, achieving selective composition presents a challenge. TFs from different families must remain compositionally distinct, even as they all recruit the same general transcription machinery, including Mediator and Pol II. Coarse‐grained simulations suggest that this is possible because TFs from different families demix into separate condensates, whereas the Pol II‐CTD co‐condenses with all of them. The underlying reason is that the interaction modes driving selective demixing both rely on aromatic residues, enabling the aromatic‐rich CTD to engage with each condensate [96]. The earlier finding that diverse activation domains all partition into Mediator condensates [33] fits within this model, in which selectivity and universality are two aspects of the same grammar. A separate question is whether this IDR‐encoded specificity actually requires condensation. Findings from Chong et al. suggest it does not: TF‐IDRs form selective, sequence‐specific interaction hubs at endogenous loci with no detectable phase separation [97]. This warrants caution, however: at this length scale a non‐phase‐separated hub may be indistinguishable from a DNA‐templated condensate in cells (see Section 6). At a minimum, the interactions that confer selectivity operate without large‐scale phase separation, which makes it difficult to attribute selectivity to condensate formation rather than to the underlying multivalent interactions. This is particularly the case since apparent selectivity can also arise without grammar at all, as in herpesvirus compartments that enrich Pol II through nonspecific protein‐DNA interactions rather than a sequence‐encoded environment [98].

The clearest evidence that selectivity matters comes from disease, where altered partitioning is itself pathogenic. A sequence change can either exclude a factor from a condensates, as seen when alanine‐repeat expansions “unblend” the TF HOXD13 from Mediator condensates in a hereditary limb malformation [99], or drive it into the wrong compartment, as seen when frameshifts that append an arginine‐rich basic tail mispartition proteins into the nucleolus across hundreds of disease‐linked variants [100]. Cancer provides the most dramatic illustration: chromosomal rearrangements fuse a DNA‐binding domain to a foreign or chimeric IDR, seeding aberrant condensates that reprogram gene expression to drive transformation. Across a broad set of such oncofusions, including the three most frequent drivers of translocation renal cell carcinoma, this gain of function converges on a shared sequence signature, an enrichment of π and π‐interacting residues, that is necessary and sufficient to partition Pol II and activate the wrong genes [95]. In each case, the pathogenic event is a change in partition localization rather than in the abundance of any factor. The grammar framework thus explains how promiscuous‐looking IDR interactions can specify composition, and selective partitioning is clearly important for gene control and disrupted in disease. What remains open is whether this selectivity also operates below the threshold of condensation: the interactions encoding it can occur without a condensate, but whether they do so to functional effect in cells is unresolved.

### 4.6 Motif Recognition by Transcription Factors

Motif recognition by TFs is fundamental to the spatiotemporal regulation of gene expression [101]. We posit that motif recognition at accessible DNA regions represents the initial step in the nucleation of transcriptional condensates. But does condensation play a role in motif recognition itself? The canonical two‐state model of protein‐DNA recognition assumes that TFs exist in a DNA‐bound or freely diffusing state. In this framework, sequence determines the frequency and lifetimes of given TF‐DNA interactions [102]. However, both in vitro and in vivo measurements challenge this simplistic view [103]. Although predicted TF motifs are abundant throughout the genome, only a small fraction exhibit substantial TF occupancy, indicating that sequence alone is likely insufficient to explain specificity [74].

From a biophysical perspective, non‐sequence‐specific interactions such as sliding and hopping along DNA are thought to contribute to motif recognition [103, 104, 105, 106]. These models and data suggest that TFs can sample a larger search space during each binding event through 1D motion, thereby increasing the efficiency of target‐site recognition. A recent study from the Ha and Wu labs found that the TF GAGA (GAF) from *Drosophila* combines both 3D and 1D diffusion to locate cognate motifs, using smFRET and optical tweezers‐based approaches [107]. Emerging evidence further suggests that the kinetics of TF association may be as important as dissociation in determining occupancy and specificity. Marklund et al. recently combined high‐throughput protein binding microarray experiments and theoretical modeling using LacI as a model system and found that sequence specificity is mainly governed by the rate of association [108]. Notably, to fit their data, the model required invoking a third “searching” phase, a non‐sequence‐specific DNA‐bound state [108]. Biologically, motif recognition is now understood to be governed by factors beyond nucleotide sequence alone. DNA shape, local chromatin environment, and cooperative interactions with cofactors all contribute to TF binding specificity and occupancy. These features have been well‐studied and reviewed [109, 110].

The interplay between TF concentration, binding dynamics, and compartment formation is central to TF specificity. In Morin et al., KLF4 was shown to form surface condensates on individual long DNA molecules (∼48.5 kbp) [42]. In this in vitro system, condensates preferentially nucleated at regions with many high affinity sites and, at higher TF concentrations, acted as energetic sinks that suppressed non‐sequence‐specific binding [42]. More broadly, biochemical studies increasingly implicate IDRs in enhancing TF specificity. Consistent with this idea, a recent study in living cells demonstrated that the specificity of the human TFs SP1 and KLF1 arises from a collective network of weak, unstructured interactions scaffolded by sequence‐specific DNA binding, using oblique line‐scan microscopy coupled with proximity‐assisted photoactivation [111]. Notably, this study did not detect micron‐scale TF foci. Nevertheless, TF self‐interactions—measured by this approach—were essential for conferring binding specificity.

Together, these observations support a more nuanced model of motif recognition in which sequence, chromatin context, molecular kinetics, and cooperative interactions collectively determine TF binding and regulatory function. There is increasing evidence that IDRs are important for this recognition and condensation may, in some instances, even help TFs distinguish clusters of binding sites within regulatory sequences from randomly occurring motifs, thereby helping sequence specificity.

## 5 Transcriptional Compartments as a Spectrum Defined by Molecular Interactions

Emerging functional evidence shows that biomolecular condensation—as opposed to the multivalent, network interactions that underlie it—is correlated with gene regulatory contexts where all‐or‐nothing transitions are required (i.e. cell‐fate, stress, environmental sensing, disease contexts). In contrast, the majority of genes are tuned across a range of expression levels in response to changes in TF dose, signal strength, and chromatin context, and the literature does not, to our knowledge, report the involvement of condensation in these graded and continuously tunable responses. How, then, is condensation integrated into our understanding of gene expression and transcriptional regulation?

TF binding to multiple sites alone—without crossing a phase boundary—is more appropriate for generating fine‐tuned functional responses in gene expression [112]. Binding/unbinding of TFs and cofactors to binding sites at enhancer and promoters can produce tunable responses. As stated above, the nucleation of biomolecular condensates are often characterized by abrupt transitions, which are not conducive to subtle functional responses. Condensates can of course respond rapidly to subtle changes in input—small fluctuations can lead to abrupt transitions—but the functional output generally remains the same. Condensation therefore appears not to be a general mechanism of transcriptional output, but a specialized one, deployed where the regulatory situation calls for decisive, step‐like behavior once a saturation concentration is crossed. Notably, this kind of switch does not seem to be invoked to tune an individual gene, but is reserved for significant transitions in cellular state. This makes sense, since the strong induction it produces is itself what can push the cell from one state into another. Based on the evidence available today, condensation appears to be associated with consequential, whole‐cell decisions rather than routine regulatory adjustments. However, too few contexts have been examined to know how broadly this pattern applies.

### 5.1 A Spectrum of Transcriptional Compartments

**FIGURE 2:**
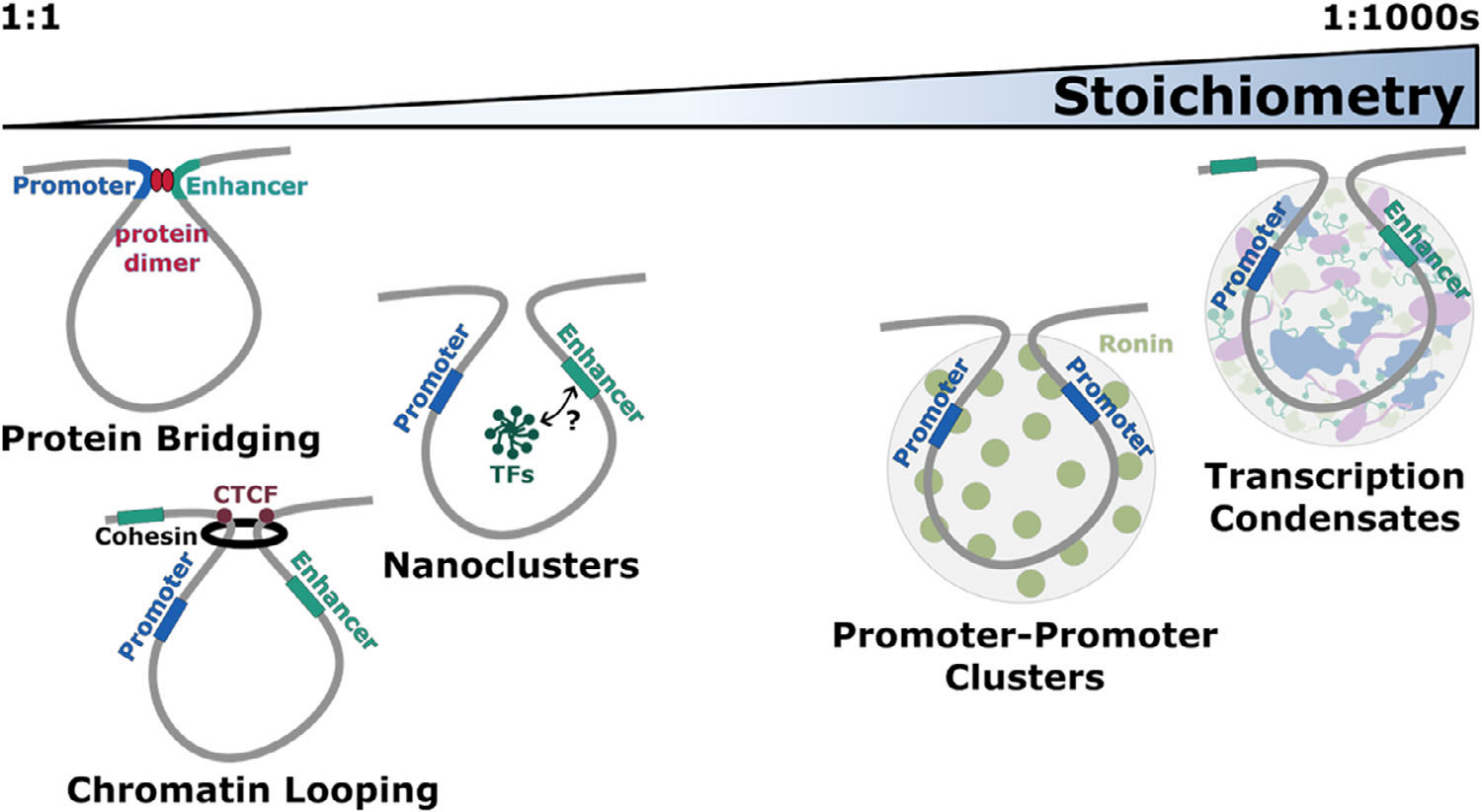
We propose that transcriptional compartments can be organized along a spectrum of stoichiometries and molecular architectures. Transcription activation is mediated by interactions spanning a wide range of assembly sizes and stoichiometries. At the lowest stoichiometry, structured protein domains form well‐defined, low‐copy‐number complexes that physically connect enhancers and promoters. Examples include protein bridges mediated by the DNA‐binding factor YY1. Cohesin and CTCF similarly form stoichiometrically defined complexes that bring regulatory elements into proximity or insulate them from neighboring genomic regions by establishing topologically associated domain boundaries. At intermediate stoichiometry, TFs have been reported to self‐assemble in solution into discrete nanoclusters. This has been shown, for example, for the oncogenic fusion protein NUP98‐HOXA9, which adopts micelle‐like nanoscale assemblies with the DNA‐binding domains (depicted as circles) being oriented toward the exterior, potentially maintaining transcriptional competence. At the highest stoichiometry, liquid‐like biomolecular condensates, such as promoter‐promoter clusters and transcriptional condensates at super‐enhancers, are reported to contain thousands of molecules and to concentrate the transcriptional machinery as well as coactivators around active regulatory DNA elements, potentially creating compositionally distinct microenvironments that facilitate gene activation.

Taken together, there is increasing evidence that certain genomic loci utilize condensates for gene regulation [27, 97] while others rely on stoichiometric interactions to spatially compartmentalize the transcriptional machinery [5]. We believe that these views are not mutually exclusive and that assemblies exist along a continuum in which stable, structured interactions coexist with dynamic, disordered ones: at one end, complexes of fixed stoichiometry built from a small number of defined contacts; in the middle, sub‐saturation multivalent clusters; and at the other end, large phase‐separated condensates of the kind reported at super‐enhancers (Figure 2).

A useful way to think about this continuum is in terms of two components: a universal structured core comprising the pre‐initiation complex (which includes Pol II) and Mediator, both assembled through stable, stoichiometric interactions at every active gene [113], and a variable, gene‐specific layer constituted primarily by sequence‐specific TFs and context‐dependent coactivators, all highly enriched in IDRs [114, 115]. It is through their multivalent, IDR‐mediated contacts with each other and with the general machinery that larger assemblies are probably nucleated. The extent of this layer, determined by the valency, concentration, and interaction strength of the recruited factors, likely dictates where a given locus falls along this spectrum. At one end, a few contacts would be expected to produce a small, well‐defined complex; additional contacts would generate sub‐saturation clusters; and beyond a critical level of multivalency, condensates would form. Which regime a gene operates in is likely a gene‐specific property, reflecting whether its regulation calls for a graded or an all‐or‐nothing response, and it is dynamically tunable as factor abundance shifts in response to developmental or signalling cues. Evidence from disease is illustrative of this logic: the oncofusions and repeat‐expansion variants discussed above (Section 4.5), together with oncogenic mutations that promote self‐association, as in NPM1 and ENL, can each push assemblies further along the spectrum than is physiological, driving aberrant condensates and gene expression programmes [99, 116, 117, 118].

In the absence of such disease‐associated perturbations, however, much of the functionally relevant regulation appears to occur across the broad middle of this spectrum, where assemblies remain poorly characterized and their stoichiometry and governing physical principles are correspondingly ill‐defined. Part of this difficulty stems from the different types of molecular interactions that contribute to these assemblies, which we discuss next.

### 5.2 Several Distinct Types of Interactions are Required to Build Transcriptional Compartments

**FIGURE 3:**
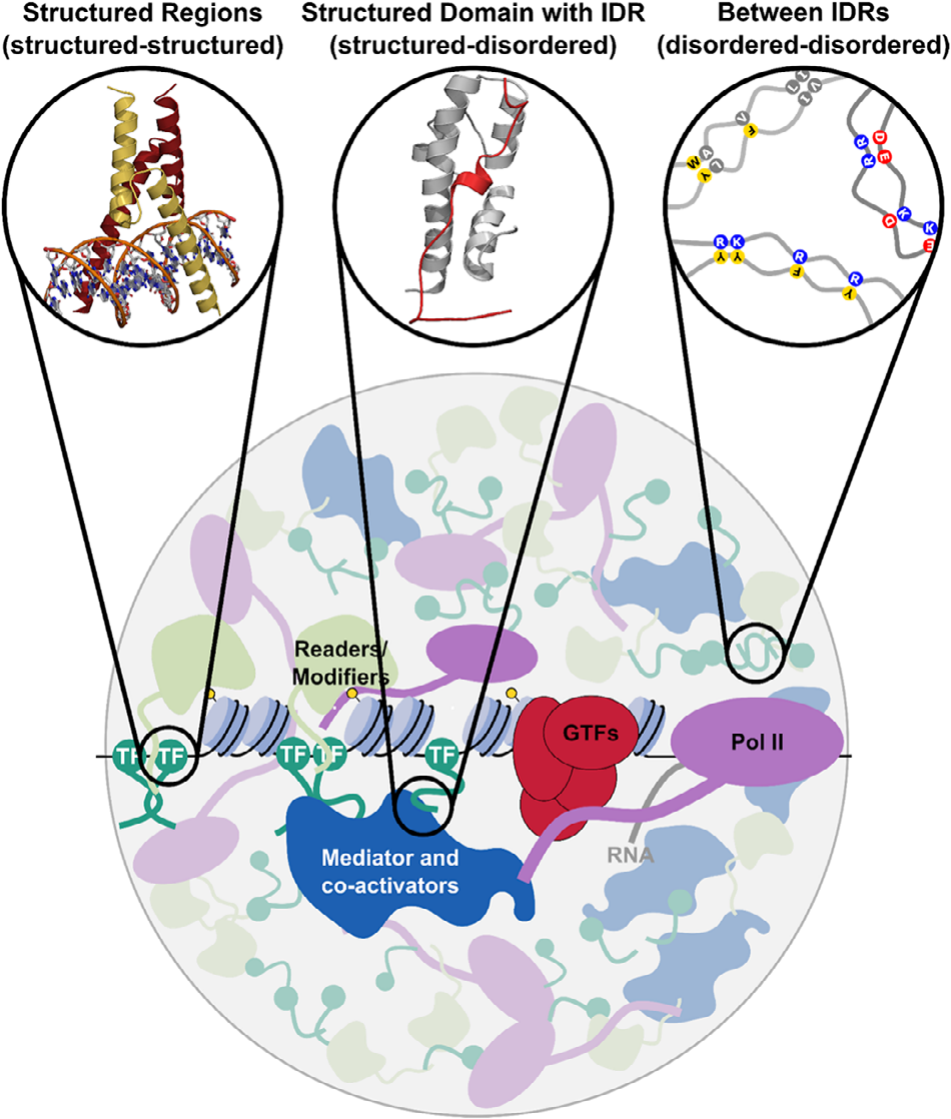
Different types of molecular interactions contribute to transcriptional compartment formation. The formation of transcriptional compartments involves multiple modes of interaction. Folded domains on TFs must recognize and bind their target DNA sequences, and in some cases, DNA‐binding domains from different TFs interact with each other. In the example shown, the TFs E47 and NeuroD1 fold together upon binding the E‐box DNA element (PDB: 2QL2). Other structured interactions include the assembly of multiprotein complexes such as Mediator or Pol II. Structured domains also frequently interact with IDRs within transcriptional compartments, as exemplified by the Mediator subunit Med15 binding to the IDR of Gcn4 (PDB: 2KO4). Similarly, the reading, writing, and erasing of epigenetic marks on disordered histone tails require interactions between folded domains and the disordered histone tails. Finally, interactions can occur between fully disordered proteins. Due to their disordered nature, conventional structures are typically unavailable for such complexes. The schematic illustrates how specific amino acid types within IDRs mediate intermolecular contacts: aromatic residues engage in π–π stacking, aliphatic residues form hydrophobic contacts, and cationic residues (particularly arginine) interact with aromatic residues through cation‐π interactions or with anionic residues through electrostatic attraction [152]. Beyond these examples, ID‐IDR contacts have been documented for the Pol II‐CTD and the IDRs of chromatin modifiers.

Even though proteins involved in transcription are substantially enriched in IDRs [114, 115], the fact that this enrichment scales with organismal complexity particularly strongly for TFs [119] shows that the basic act of transcription does not require IDR‐mediated interactions, but is consistent with the idea that they enable the flexible, combinatorial regulation that complex genomes require. Already from the outset, both structured and IDR‐mediated interactions contribute to compartment formation (Figure 3): individual TFs bind DNA through structured domains and recruit cofactors and the general machinery through their IDRs using a combination of IDR‐IDR and structured‐IDR interactions [120].

Interactions between structured domains provide the stable, high‐specificity contacts that assemble the core machinery. Thanks to roughly five decades of atomistic structural work, we now have a detailed mechanistic understanding of how these interactions drive selective and stable complex formation through shape complementarity and a combination of hydrophobic, electrostatic and hydrogen‐bonding contacts. In this way, large molecular machines such as Pol II and Mediator are assembled reproducibly, and it is the precision of these interfaces that makes the enzymatic activities of the transcriptional machinery possible [113].

Interactions between structured domains and IDRs couple the stable core to flexible, gene‐specific regulation, and operate through two distinct mechanisms. In coupled folding‐upon‐binding, a disordered motif within an IDR adopts a defined conformation when engaging a folded domain. For example, the 9aaTAD motif, which is found in the IDRs of some TFs such as p53 and Gal4 forms amphipathic helices when binding structured coactivator domains [121], and the disordered activation domains of c‐Myb and CREB fold into helices upon binding the structured KIX domain of CBP/p300 [122, 123]. Bromodomains and chromodomains similarly recognize acetylated and methylated lysines within the disordered tails of histones through well‐defined binding pockets [124]. In fuzzy complexes, by contrast, the IDR partner remains dynamic while making distributed contacts with the folded surface: acidic activation domains in TF IDRs engage the structured activator‐binding domains of Med15 through multiple transient contacts, remaining disordered throughout [125, 126, 127]. Both mechanisms allow compartment composition to be rapidly and reversibly tuned, since the short motifs involved are frequently sites of post‐translational modifications that create or destroy binding interfaces [128].

Interactions between IDRs supply the weak, multivalent contacts that are known to drive condensation of larger and more dynamic compartments. The key feature of these interactions is multivalency: individually weak contacts accumulate through avidity, yielding assemblies with high apparent affinity and slow effective off‐rates while remaining dynamic at the level of individual contacts [92]. In transcriptional compartments specifically, a range of interaction types have been reported: acidic residues across TF activation domains engage the disordered C‐terminal region of MED1 through electrostatic contacts [33], while the FUS‐IDR engages the Pol II‐CTD through a broader mix of residue types and interaction modes, including hydrophobic, π‐stacking and cation‐π contacts [129]. The selectivity of IDR‐IDR interactions appears to depend on sequence composition, with computational work suggesting that different TF families show distinct preferences based on their aromatic, charged and aliphatic residue content [96]. These interactions are most associated with condensation but are also likely to function in transcriptional regulation whether or not a phase boundary is crossed [63].

In any transcriptional compartment these three types of interactions likely act in concert: structured domains anchor TFs to DNA and assemble the core machinery, folded‐domain motif contacts recruit specific cofactors, and IDR‐mediated multivalency nucleates a broader assembly, with transcriptional output emerging from the combination. Studying any one type in isolation therefore, yields an incomplete picture. Understanding how transcriptional compartments are regulated will therefore require methods that can capture multiple interaction types simultaneously, across the full range of stoichiometries from defined complexes to condensates, and it is the limitations of the current methodological toolkit, as much as any conceptual gap, that stand in the way. We turn next to the experimental and computational approaches available for this task, and to where their principal challenges lie.

**FIGURE 4:**
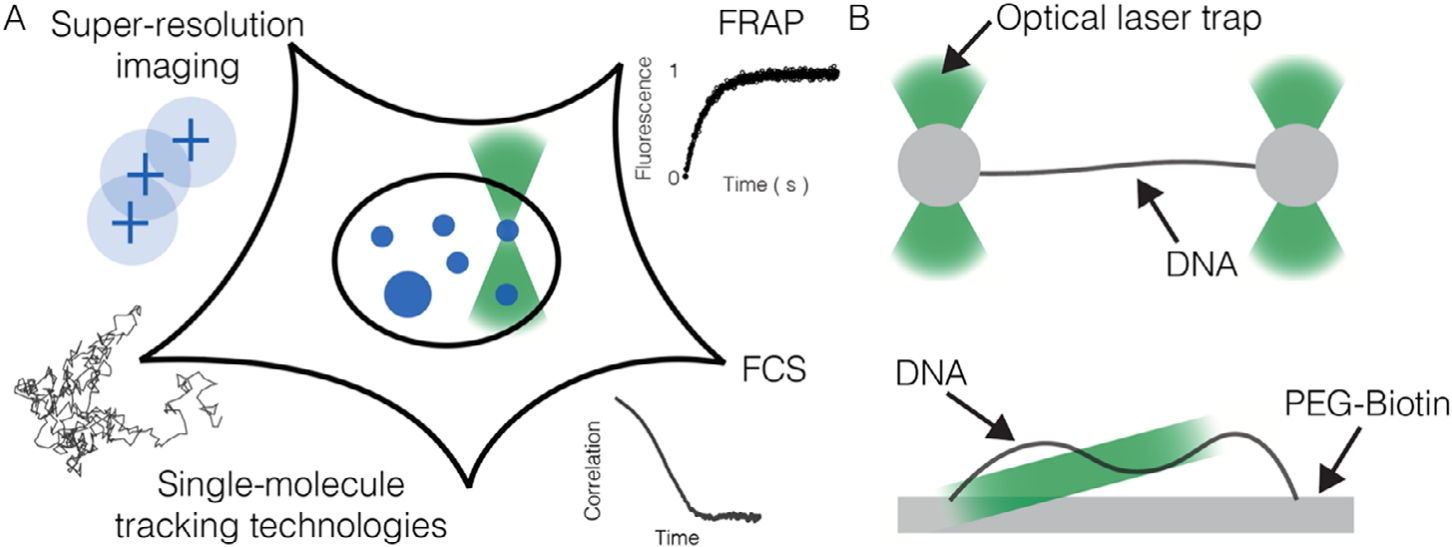
State‐of‐the‐art methodologies to investigate and image transcriptional compartments. (A) Fluorescence‐based imaging technologies to investigate transcriptional compartments, including super‐resolution imaging, fluorescence recovery after photobleaching (FRAP), fluorescence correlation spectroscopy (FCS), and single‐molecule tracking technologies. (B) Single molecule biophysics approaches, including correlative optical tweezers/fluorescence‐based imaging and DNA curtain/carpet assays that use HiLO TIRF microscopy to image the interactions of proteins with DNA.

## 6 State of the Art Methods in the Study of Transcriptional Compartments

The biological diversity of transcriptional compartments and the consequences of their dysregulation in disease underscores the importance of understanding how these assemblies form, what physical properties they possess, and how their composition determines their function. Yet, despite the growing number of biological contexts in which they have been implicated, fundamental questions about their very nature remain unresolved. How are thermodynamic boundaries established by collections of proteins in the cell? Once nucleated, what are the turnover and exchange kinetics within the transcriptional compartment and how is this linked to function? What are the stoichiometries of these compartments and how do these compartments generate force? And lastly, how does the molecular grammar of IDR‐mediated interactions drive the assembly and disassembly of such organizing centers?

In this section, we will discuss the state‐of‐the‐art technologies that can be used to tackle these questions and discuss their limitations and where we need, as we see it, to devote resources and commitment toward developing new technologies or where there are already opportunities for development and interdisciplinarity.

### 6.1 How are Thermodynamic Boundaries Established?

In vitro analyses of bulk droplets formed using one‐ or two‐component  systems enable the experimental determination of thermodynamic boundaries that define phase‐separated and mixed states based on concentrations and interaction strengths. However, these boundaries are “blurrier” in cellular environments, where condensates are multi‐component systems that may include hundreds of different proteins, nucleic acids, and a wide range of post‐translational modifications. The cell is out of equilibrium and the condensates consume energy, further complicating the interpretation of phase behavior and challenging simple thermodynamic descriptions derived from minimal systems. What can be measured in cells is the dilute‐phase boundary: by quantifying the concentration of a fluorescently tagged protein at the onset of condensate formation, an apparent intracellular saturation concentration can be determined, and this has been done for several endogenous condensate systems [130]. Full binodals, which additionally require the dense‐phase concentration, are far harder to obtain in cells, and indeed the same work showed that a single fixed saturation concentration is often not even a valid description in the multicomponent cellular environment. The most quantitative boundary measurements, therefore, remain accessible only in vitro. Below, we first describe how full phase diagrams are drawn in reconstituted systems, then turn to the assembly states that populate the sub‐saturated regime, where in‐cell measurements become possible.

#### 6.1.1 In Vitro Phase Diagrams

Phase diagrams provide a framework for describing how molecular systems transition between homogeneous and phase‐separated states as a function of variables such as concentration and environmental conditions. The binodal defines the boundary between these states and describes the compositions of the coexisting dilute and dense phases. In studies of biomolecular condensates in vitro and in cells, many groups focus on determining the saturation concentration, which corresponds to the dilute phase boundary above which phase separation occurs. While this provides essential information about when phase separation happens, complete binodals that define both dilute and dense phase concentrations offer deeper insight into why it occurs and how condensates behave. Measuring both arms of the binodal reveals the strength of the molecular interactions that drive assembly formation: a wider two‐phase region indicates stronger net attraction between molecules, while the dense phase concentration reflects the internal organization that determines condensate properties such as viscosity and how rapidly molecules exchange with the surrounding environment. For mixtures, quantifying both phase compositions reveals whether self‐interactions or cross‐interactions between different components dominate assembly. These measurements therefore allow a transition from qualitative statements that a TF or coactivator “can phase separate” to mechanistic descriptions of how strongly it self‐associates, how far below its nuclear concentration the phase boundary lies, and whether it co‐assembles with partners or instead recruits them into a pre‐existing condensate. However, complete binodals of this kind have, as far as we are aware, only rarely been determined for transcriptional components. Obtaining complete phase diagrams remains experimentally challenging, particularly for the dense phase where high protein concentrations complicate quantification.

Importantly, most in vitro measurements of phase behavior are performed under controlled conditions that allow the system to approach equilibrium, whereas condensates in cells exist in a dynamic environment that is better described as a non‐equilibrium steady state. Cellular processes including transcription, molecular turnover, enzymatic activity, and active transport can influence condensate formation, composition, and material properties, meaning that equilibrium phase diagrams provide an important but incomplete description of cellular condensates. Understanding how the parameters measured in vitro relate to dynamic behavior in cells remains a major challenge. Nevertheless, these approaches have provided key insights into the molecular interactions governing condensate formation, including the contributions of self‐association, multivalent interactions, and partner‐dependent assembly, and have helped establish links between condensate material properties and biological function. The following paragraphs describe the key methodological approaches used to measure these properties and what they have revealed about transcriptional condensates.

Determination of phase diagrams by centrifugation and analytical quantification can be done in any laboratory as it does not require specialized equipment [131]. Here, protein solutions are prepared at defined total concentrations under fixed conditions, allowed to phase separate, and then centrifuged to separate dilute and dense phases. The supernatant (dilute phase) and pellet (dense phase) are collected and their protein concentrations quantified by spectroscopic or colorimetric assays, providing paired points that define both arms of the binodal. This label‐free approach works for many proteins but demands large samples, can perturb soft or small condensates during handling, and is laborious to extend across large phase spaces.

inPhase is a microscopy‐based volume‐conservation method in which phase diagrams are constructed from imaging of condensates in well‐defined sample geometries [132]. By measuring the volume fraction of the condensed phase from a known total protein amount, mass conservation yields both binodal arms. It is highly material‐efficient, works with or without fluorescent labels, and pairs readily with automated imaging, though its accuracy depends on robust condensate segmentation.

High‐throughput microfluidic methods, so‐called PhaseScan, generate libraries of nanoliter droplets that each contain a distinct combination of protein and cosolutes, and then classify individual droplets as single‐phase or phase‐separated via imaging [133]. Scanning composition space this way delineates boundaries across multidimensional spaces (protein, salt, ligand) at very low per‐condition sample consumption, making large phase spaces feasible to map [134]. However, they require specialized microfluidic hardware and non‐trivial expertise to implement and maintain. Moreover, the extraction of quantitative dilute and dense phase concentrations requires fluorescent labelling of the molecules.

Q‐phase is an imaging‐based method that reconstructs phase diagrams by exploiting refractive index differences between coexisting phases [135]. It uses quantitative phase imaging, which records how strongly the light wave is delayed in different parts of the sample; after calibration, this gives a map of protein concentrations, yielding the full binodal without labels or droplet segmentation. This approach is robust to complex morphologies because it does not rely on intensity‐based thresholding of condensates. Limitations include the need for specialized quantitative phase imaging instrumentation, accurate refractive index‐concentration calibration, and careful control of equilibration and imaging conditions.

Across these methods, rigorous full‐binodal determination has so far rarely been turned on transcriptional components, even though TFs and coactivators are among the proteins most heavily characterized by simpler condensed‐fraction and turbidity assays that establish only whether phase separation occurs. To our knowledge, inPhase is the only method in this group which has directly been applied to a transcriptional protein. Here, the full temperature‐dependent phase diagram of the chromatin reader BRD4 was mapped in vitro, yielding a dilute boundary near 2 µM, dense phase three orders of magnitude higher [136]. On its own the binodal is a static description, but its real strength lies in the combination with cell biology and theory: super‐resolution imaging showed BRD4 foci that fail to coarsen as equilibrium phase separation would predict, and an active field theory explained this as non‐equilibrium size control. Cases like this argue that progress on transcriptional compartments will increasingly depend on interdisciplinary work. Applying the methods described in this section, ideally in combination with other disciplines such as in‐cell imaging and theory, is, in our view, among the clearer near‐term opportunities in the field.

#### 6.1.2 Methods to Study Nanoscale Assemblies that Form Below c_sat_

Below the saturation concentration, the dilute phase is not necessarily featureless. For some phase‐separating proteins, a fraction of the protein assembles into small, heterogeneous nanoscale clusters rather than remaining monomeric, and these are increasingly recognized as distinct assembly states that precede or accompany condensate formation [137]. Whether this is a general property of phase‐separating proteins or is restricted to certain sequences, and how sub‐saturation clustering relates to macroscopic phase separation, remain open questions. For a TF, this distinction could be functionally important. Binding sites at enhancers and super‐enhancers are themselves often clustered and a single monomeric TF can occupy only one site at a time, whereas a multivalent cluster could potentially read several sites at once, leading to engagement with higher avidity and holding occupancy that isolated low‐affinity contacts could not. Whether TFs typically arrive at DNA pre‐assembled into such clusters is therefore directly relevant to how target sites are selected.

However, sub‐saturation clusters remain a relatively new area of investigation with few systematic studies to date. Here, we provide an overview of the techniques that have been employed (Figure 4A). These nanometer‐ to submicrometer‐sized species can be detected using several complementary approaches, each resolving a different property: dynamic light scattering (DLS) reports on size distributions in solution; mass photometry provides single‐particle mass measurements, resolving cluster stoichiometry; FCS and single‐molecule FRET (smFRET) report on diffusion, molecular interactions, and conformation; super‐resolution and single‐molecule fluorescence imaging provide access to spatial organization; and electron microscopy (EM) resolves the morphology of nanoscale assemblies directly.

To date, such assemblies have been studied almost exclusively in vitro. DLS of FUS and related FET family proteins (TAF15, EWSR1) revealed heterogeneous clusters of ∼7 to 100 nm in subsaturated solutions, as confirmed by transmission EM [138]. Mass photometry demonstrated that α‐synuclein forms nanoscale clusters containing tens to hundreds of molecules at physiological concentrations. Remarkably, these clusters form instantaneously even under conditions where macroscopic droplets take days to appear [134]. Freeze‐fracture deep‐etch EM showed that nuclear speckle proteins including SRSFs and TDP‐43 form size‐limited assemblies of 23–45 nm, each containing ∼20‐50 molecules. Interestingly, the exact size and architecture depends on attractions and repulsions between the different protein domains [139]. FCS and smFRET showed that the oncogenic fusion protein NUP98‐HOXA9 forms concentration‐dependent nanoclusters with micelle‐like organization, where the DNA‐binding domain is oriented toward the exterior, potentially facilitating chromatin interactions. These nanoclusters may be more physiologically relevant than large macroscopic droplets, which emerge only at high protein concentrations [140].

Direct observation of such assemblies in cells is rarer but emerging. In *Drosophila* embryos, lattice light‐sheet microscopy revealed that the TF Bicoid forms transient high‐density hubs that facilitate DNA binding, which is particularly important in the posterior where Bicoid concentrations are low [141]. This suggests that one function of these small assemblies is to facilitate low‐affinity interactions. In yeast, super‐resolution PALM imaging showed that G1/S factors (Swi4, Mbp1, Swi6) assemble into discrete ∼8‐molecule clusters directly at DNA promoters [142]. In the coming years, it will be exciting to see whether these nanoscale assemblies are a general feature of TFs or whether only certain classes can form them. Furthermore, since numerous TFs have been reported to phase separate in bulk and/or condense on DNA, are these assemblies always an intermediate state on the way to larger condensates, as observed for the Nup98‐HoxA9 fusion protein in solution [27, 33, 42, 43, 96, 97]? However, it is unclear whether the biophysics underlying the formation of these nanoscale assemblies studied in vitro is relevant for these clusters observed in cells remains to be seen.

### 6.2 What are the Turnover and Exchange Kinetics Within Transcriptional Compartments?

Transcriptional compartments are small and highly dynamic, typically turning over on time scales of tens of seconds, though this varies widely between compartment types. The biophysical mechanisms that set these time scales, and how they connect to function, remain largely unclear. The clearest evidence that turnover matters comes from individual factors: at the *GAL* genes in budding yeast, the dwell time of the activator Gal4 sets the size of the transcriptional bursts it drives [143]. Whether the surrounding compartment shapes such dwell times is far less settled, and clustering of a single factor has been reported both to drive transcriptional bursting [144] and to speed up target search without activating transcription [73]. One reason to expect an effect is material state: a compartment's viscosity should govern how quickly molecules move through it and therefore how long they remain bound. Transcriptional condensates differ sharply here, from the liquid‐like coactivator assemblies of BRD4 and Mediator, which recover within seconds by FRAP [27], to the gel‐like NDF‐FACT condensates that recover only partially over minutes, in keeping with their role as processive machinery that travels with elongating Pol II [145]. Untangling how turnover is regulated, and how it shapes output, thus rests on directly imaging the dynamics of compartments and of the molecules within them.

#### 6.2.1 Bulk Measurements in Living Cells: FRAP and FCS

TFs interact with DNA and chromatin in a highly dynamic and transient manner. Owing to its relative ease of implementation, fluorescence recovery after photobleaching (FRAP) became a widely used method in the early 2000s for studying TF dynamics [146]. FRAP measures the recovery of fluorescence within a photobleached region and has been extensively applied to estimate residence times, diffusion coefficients, and exchange rates of TFs, other chromatin‐associated proteins, and chromatin itself within the nucleus [101]. Applied to transcriptional compartments, FRAP of the coactivators BRD4 and MED1 showed fluorescence recovery on the timescale of seconds [27]. FRAP measurements of Pol II and Mediator within transcriptional compartments in mouse embryonic stem cells revealed somewhat slower recovery, over tens of seconds [28]. Notably, these compartments did not exhibit complete fluorescence recovery, suggesting the presence of a relatively immobile or stably bound fraction of both complexes [28]. Together, these observations indicate that transcriptional compartments are highly dynamic structures.

Fluorescence correlation spectroscopy (FCS) provides a complementary approach for probing molecular dynamics. Rather than monitoring fluorescence recovery, FCS analyses fluctuations in fluorescence intensity within a small confocal volume and quantifies their temporal correlations. By fitting these correlations to appropriate mathematical models, it is possible to extract diffusion coefficients, oligomerization states, and local molecular concentrations [147]. A DNA‐bound TF, for example, moves more slowly and therefore produces more sustained temporal correlations than a freely diffusing TF moving through the confocal volume. FCS has been widely applied to investigate TF behavior, including Sox2 DNA binding in mouse embryos [148], Stat3 oligomerization [149], and glucocorticoid receptor (GR) oligomerization [150], among others. It has also been used to characterize synthetic transcriptional compartments: tethering a TAF15 low‐complexity domain to a LacI array, Chong et al. measured local concentrations orders of magnitude above those of a LacI‐only control [97]. Beyond cells, FCS has been applied to understand the dynamics of individual factors in vitro in reconstituted bulk droplets composed of linker histones [67] and Laf1 droplets [151].

#### 6.2.2 One Molecule at a Time: Single‐Molecule Tracking and Live‐Cell Super‐Resolution Microscopy

FRAP and FCS are bulk approaches that require averaging over many molecules and model fitting to extract biophysical parameters. Single‐molecule tracking (SMT) instead follows individual proteins directly [152]. By combining sparse labelling with bright, photostable dyes and illumination schemes such as highly inclined and laminated optical sheet (HiLO) or TIRF microscopy, SMT resolves bound and diffusing molecules across the nucleus, yielding two central observables: the residence time a molecule remains bound and its diffusion coefficient while mobile. The distribution of residence times is itself informative, separating brief, exploratory contacts from the stable, likely‐productive binding events. Across diverse sequence‐specific TFs, including the pluripotency factors Sox2 and Oct4 [153], the pioneer factor FoxA1 [154], and the glucocorticoid and estrogen receptors [155], specific‐site engagement is transient, with residence times of order seconds. The shape of these distributions is also telling. Rather than the two discrete states, non‐specific and specific, of the classical bi‐exponential model, several mammalian factors instead show a continuous, power‐law‐like distribution [156]. This behavior may reflect higher‐order protein‐DNA clustering, in which a range of complex sizes produces a continuum of dwell times [157].

The same approach has been scaled to the transcription machinery as a whole. A recent study from the Wu lab has applied SMT to dozens of proteins across Pol I, II, and III machineries in living yeast [158]. It revealed striking differences: while the Pol I and III pre‐initiation complexes (PICs), which sustain constitutive rRNA and tRNA synthesis, engage chromatin in long‐lived interactions, the Pol II PIC and its associated regulators exchanged rapidly with the nucleoplasm. Because transcriptional compartments are small and short‐lived, however, resolving their internal organization demands spatial resolution beyond the diffraction limit (∼200 nm) and temporal resolution on msec time scale.

Super‐resolution imaging approaches such as direct stochastic optical reconstruction microscopy (dSTORM) or photoactivated localization microscopy (PALM) on fixed samples have successfully characterized the size and morphologies of transcriptional compartments in many different biological contexts [159]. However, fixed‐sample imaging captures structure without dynamics. The key advance was to extend super‐resolution imaging to living cells. Over the past decade, work from the Cissé lab using time‐correlated PALM (tcPALM) revealed that Pol II and the Mediator complex form transient, diffraction‐limited clusters in mouse embryonic stem cells [28, 29]. From this approach, they obtained estimates of the average cluster size (∼40 nm)  and dynamic lifetimes of such clusters (∼10 s). Using similar approaches, the same group linked dynamics to output: using an MS2‐based mRNA reporter to follow nascent transcription in real time, they found that cluster lifetime correlates with the amount of mRNA subsequently produced [31]. Live‐cell super‐resolution has thus established that transcription occurs within highly dynamic Pol II clusters whose lifetimes track transcriptional activity, although whether cluster formation and dissolution *cause* bursts, rather than being co‐regulated with them, remains debated (see Section 4.2).

A new technology that has the potential to reshape this area is minimal photon flux (MINFLUX) microscopy, which localises single fluorophores by fast scanning of patterned excitation beams. MINFLUX enables tracking of individual molecules with a temporal resolution of 1 ms and a spatial resolution of 1–3 nm [160]. This level of precision provides a powerful opportunity to resolve how individual TFs explore, engage with, and exit transcriptional compartments on shorter time and spatial scales. To date, however, its successes have come in other systems such as by providing key dynamic insights into motor proteins in cytoskeletal systems [160]. Current development focuses on integrating two‐color tracking, which would enable conformation and position to be read out simultaneously and could be especially powerful for dissecting TF IDRs or Pol II activity. While MINFLUX and related super‐resolution approaches provide unprecedented insight into the dynamics of individual molecules, translating these nanoscale observations into mechanistic insights of transcription compartmentalization ultimately requires information on the conformational space of TFs and how this is linked to functional relevant biological contexts. To our knowledge, MINFLUX has successfully been employed to study the sub‐diffusive motion of chromatin in living cells [161], and, as yet, represents only a promising direction for the study of TF binding and tracking.

### 6.3 What are the Molecular Stoichiometries of Transcriptional Compartments and How do They Generate Force?

Where a transcriptional compartment sits between a defined complex and a condensate is, at bottom, a question of composition: a complex holds a fixed, low ratio of protein to DNA, a condensate a high ratio. Locating a compartment on this spectrum means measuring that ratio directly — how many protein molecules associate with how much DNA. Recent work has shown that such condensates have an additional, less obvious property: as they gather DNA, they exert measurable mechanical force on the strand, and can even pull DNA in, thereby changing that ratio itself. What that force is for remains unknown, but one possibility is compelling: by drawing distant elements such as an enhancer and its promoter together, it could help them engage, which would speak directly to the unresolved question of enhancer–promoter communication. Force and composition therefore cannot be treated in isolation, and both are needed to characterize the state of a compartment. Neither is readily measured in living cells, where the distribution of factors and the amount of co‐condensed DNA cannot yet be read out together and nuclear force scales remain largely unknown. Most quantitative insight has instead come from reconstitution, where single‐molecule imaging of long dsDNA templates (∼10–50 kb) makes the collective behavior of many proteins tractable at the scale of an enhancer or promoter [4].

Three main assays are used in these contexts: DNA‐curtain‐based assays, DNA carpet assays, and correlative optical tweezers combined with fluorescence microscopy (Figure 4B). DNA‐curtain‐based approaches pioneered by Eric Greene and Sy Redding integrate lithographic techniques to immobilize long pieces of dsDNA on supported lipid bilayer surfaces, which are then imaged with total internal reflection fluorescence (TIRF) microscopy [162]. The DNA carpet assay is a close variant but dispenses with both the fluid bilayer and the barriers: DNA is tethered at high density to a static, PEG‐passivated surface bearing biotin anchors, giving many molecules per field of view but at random rather than registered positions [163]. Correlative optical tweezers, by contrast, tether a single linearized dsDNA molecule between two optically trapped beads and combine force measurement with confocal fluorescence imaging, so that protein binding can be observed while force is applied or recorded [164]. Throughput for both the curtain and the carpet assays is much higher than the optical tweezers‐based approach, enabling the testing of multiple conditions, and molecule counting across different concentrations is well suited to them as TIRF or highly inclined and laminated optical sheet (HiLO)‐TIRF approaches reduce most of the background fluorescence [43]. However, in these systems, force measurements are noisy and heterogeneous—optical tweezers are far more precise, as these measurements are based on sensitive displacements.

Recent years have seen the emergence of single‐molecule approaches that apply these methods to transcription compartmentalization. These approaches have provided insights into the sequence‐specific condensation of Klf4 and condensation forces by FoxA1 and Sox2 [42, 43, 47]. For Klf4, these measurements showed that it condenses on DNA near its physiological concentration (∼100 nM), which is well below the concentration needed for phase separation in solution [42]. Physically, this condensation occurs through a prewetting‐like surface transition: sequence‐guided adsorption locally concentrates the protein on the DNA, seeding condensates at positions set by the underlying sequence. To distinguish between adsorption and condensation, a careful quantification of the protein‐to‐DNA ratios was essential. FoxA1 and Sox2 also form condensates on DNA, likely through the same surface mechanism, though this has not been dissected as carefully [43, 47]. The same correlative approach quantified the forces that co‐condensation exerts on DNA: FoxA1 buffers DNA at a sub‐piconewton force of ∼0.2 pN [43], whereas Sox2 generates forces up to ∼7 pN [47], an order of magnitude higher, with its disordered regions required for force generation even though its structured DNA‐binding domain alone suffices for condensation. Critically, Sox2 also revealed how chromatin tunes this output: nucleosomes preferentially recruit Sox2 yet abolish its measurable force, acting as mechanical sinks that buffer the tension a TF condensate can impose on the genome [47]. More complicated in vitro reconstitutions combining more components of the transcriptional machinery is a long‐term goal in this area, as well as the incorporation of active lysate‐based systems, which has already been demonstrated in DNA damage repair‐based contexts [165]. The deeper challenge, however, is to make these same measurements of protein‐DNA stoichiometry and compartment forces in living cells, which has remained out of experimental reach to date.

### 6.4 What is the Molecular Grammar of IDR‐Mediated Interactions, and How Does it Govern Their Dynamics?

Understanding how IDRs drive the formation and dynamics of transcriptional compartments requires identifying the molecular rules—or “molecular grammar”—that govern their interactions. Unlike structured domains that interact through well‐defined interfaces, IDRs engage in transient, multivalent contacts that are highly dynamic and often heterogeneous [92, 166]. Without a fixed interface, the rules of IDR binding and how those contacts behave in time, how transient or readily switched they are, become two aspects of one question. Establishing these rules, for contacts both between IDRs and between IDRs and structured domains, is what would explain how transcriptional compartments achieve specificity, assembling the right factors at the right genes, from interactions that individually appear weak and promiscuous [95, 167]. Deciphering this molecular grammar therefore demands experimental and computational approaches capable of capturing both transient interactions and conformational ensembles across multiple spatial and temporal scales. This is inherently difficult, and becomes more so with the number of interacting partners, which in the crowded nuclear environment is large. The full combinatorial complexity of these contacts is therefore out of reach of any current method, and cannot be accessed at residue level in cells at all. Much of what is known about the grammar has consequently been learned from reconstituted or simulated systems, with few examples in cells, as described in the following paragraphs.

#### 6.4.1 Capturing Disorder: NMR

For the formation and function of transcriptional assemblies, all types of interactions between IDRs and structured domains are required. X‐ray crystallography and cryo‐electron microscopy have been transformative here, resolving the transcriptional machinery in extraordinary mechanistic detail, from the catalytic core of Pol II to Mediator engaged with the pre‐initiation complex [124, 168, 169, 170]. These methods are, however, suited to order: they resolve folded complexes beautifully but leave the disordered parts of those same complexes far less characterized. Capturing IDR contacts requires methods that work in the disordered state itself.

Here, nuclear magnetic resonance (NMR) is the principal experimental method for characterizing interactions between IDRs and structured surfaces, giving mechanistic insight. For example, NMR chemical shift perturbation mapping combined with paramagnetic relaxation enhancement (PRE) experiments revealed that the acidic activation domains of the yeast TFs Gcn4 and Gal4 bind a structured domain on Med15 through a “fuzzy” mechanism: hydrophobic residues gain transient helicity upon binding while sampling multiple orientations, explaining how diverse activation domains can all recruit Mediator despite lacking sequence similarity [127]. Disorder can also build directional switches: NMR showed that the regulator CITED2 irreversibly displaces HIF‐1α from CBP/p300 despite equal binding affinity, because the flexibility of both disordered partners permits a transient ternary intermediate that resets the complex in one direction only, ensuring rapid, unidirectional termination of the hypoxic response [171]. Furthermore, NMR is one of the only experimental methods with which specific interactions between IDRs themselves can be probed and intermolecular PRE experiments in particular can detect transient IDR‐IDR contacts. For instance, measurements on the IDR of EWSR1 revealed specific intramolecular contacts that bias the conformational ensemble toward states productive for self‐association [172]. In another example, NMR was combined with smFRET to show that the highly charged linker histone H1 and its chaperone prothymosin‐α form an ultrahigh‐affinity complex while both proteins remain fully disordered [173, 174]. This interaction, driven purely by the large opposite net charges of the two proteins, enables the rapid deposition and removal of H1 from chromatin required for transcriptional regulation [175]. This result demonstrated, for the first time, that high‐affinity binding can occur between fully disordered proteins, challenging the traditional view that such interactions require structured interfaces.

#### 6.4.2 Resolving IDR‐IDR Interactions: Fluorescence and Mass Spectrometry Methods

To resolve IDR‐IDR interactions, several complementary methods can provide crucial insight, albeit at lower resolution. smFRET can reveal coexisting conformationally distinct subpopulations in monomeric IDPs. It is particularly powerful when combined with other methods such as FCS, as this allows the study of dynamic networks within condensates. Ruan et al. characterized the chimeric oncogenic TF NUP98‐HOXA9 using confocal smFRET, FLIM‐FRET, and FCS, demonstrating that the disordered FG domain undergoes a dramatic conformational expansion from highly compact in the monomeric state to near‐random coil in condensates as intramolecular contacts convert to intermolecular ones during assembly [140]. Using a similar integrated approach, a study on the FUS‐IDR used smFRET to access both dilute and condensed phases: in the monomeric state, two distinct structural subpopulations were resolved, while measurements within condensates revealed chain expansion and a ∼400‐fold slowing of diffusion, quantifying the viscoelastic properties that distinguish liquid condensates from solutions [176].

Cross‐linking mass spectrometry (XL‐MS) takes a different approach, using chemical crosslinkers to covalently join proximal residues, capturing the spatial relationships present at the moment of reaction, including transient populations, and providing distance constraints that can be used for modeling. XL‐MS combined with cryo‐EM revealed that the large MED13‐IDR functions as a conformational switch: upon nuclear receptor binding, this IDR repositions to allow Pol II recruitment to Mediator [177]. The structural change appeared only as diffuse density in cryo‐EM alone. In a particularly large‐scale application, an impressive 1200 crosslinks were generated within the 52‐protein yeast Mediator‐containing pre‐initiation complex, defining the complete path of the Pol II‐CTD through the complex and positioning the entirely mobile TFIIK kinase module [170].

#### 6.4.3 Deriving the Rules: Molecular Dynamics Simulations

Molecular dynamics simulations, both atomistic and coarse‐grained, have been central to understanding IDR interactions where experiment cannot reach residue‐level detail. For instance, all‐atom simulations of complete nucleosomes captured spontaneous DNA breathing, unwrapping, and sliding at atomistic resolution, identifying how the disordered histone tails mediate DNA end reconfiguration through specific residue contacts [178]. For coarse‐grained approaches, the sticker‐spacer framework has been foundational. Developed by Pappu, Mittag, and colleagues, it established that aromatic residues, especially tyrosine, act as “stickers” whose valence and patterning determine both the propensity for phase separation and whether resulting assemblies remain liquid or mature into solid aggregates [179, 180]. Recent simulations of TF families have generalized this framework, identifying four sticker classes (aromatic, aliphatic, cationic, anionic) and revealing that condensation can be driven by two orthogonal forces: hydrophobic interactions (π–π and aliphatic contacts) or electrostatic/cation‐π interactions (particularly arginine‐tyrosine) [96]. This broader molecular grammar accounts for the diversity of TF condensate properties while explaining how the Pol II‐CTD universally partitions into all TF condensates regardless of their dominant interaction mode. It also raises the central open question: if all TF condensates share this grammar, how is any specificity achieved? Part of the answer lies in post‐translational modification. Multi‐scale simulations combined with in vivo experiments in *C. elegans* showed that the Pol II‐CTD phosphorylation state dictates both the stability and internal organization of Pol II condensates: distinct phosphorylation levels generate coexisting phases that partition into partially engulfed sub‐environments, potentially corresponding to initiation and elongation compartments, each selectively recruiting the factors appropriate to its transcriptional stage [181].

Taken together, a coherent grammar is beginning to emerge: multivalent, fuzzy contacts without fixed interfaces, the capacity for high‐affinity binding even between fully disordered partners, and a sticker‐spacer logic in which residue identity, valence, and patterning tune both phase behavior and material state. What remains least resolved is how this shared chemistry yields specific compartments rather than one indiscriminate phase, with post‐translational modification currently the most promising explanation, and whether a grammar inferred largely from reconstituted and simulated systems holds in the far more complex environment of the nucleus.

## 7 Discussion

Transcription is spatially organized into distinct foci, but neither of the models most commonly invoked to explain their formation is fully consistent with the evidence. Equilibrium LLPS predicts droplets that coarsen over time, yet the compartments described here are multi‐component, small, and transient, and where this has been tested they fail to coarsen as equilibrium phase separation would predict, remaining instead at finite size through active, non‐equilibrium processes. A model built purely on stable, structured protein bridges is similarly incomplete, since imaging studies show transcriptional assemblies with variable, often large numbers of molecules — far exceeding the fixed, low‐copy‐number stoichiometry a structured bridge would produce.

Condensation carries a clear functional signature only where regulation demands all‐or‐nothing transitions—cell‐fate decisions, stress responses, environmental sensing, disease—while the graded, tunable majority of genes shows none. This suggests that whether a given locus phase separates is, for most genes, the wrong question to ask: functional condensates should be expected preferentially at switch‐like loci, and forcing condensation onto a gene that is normally tuned continuously should degrade its tunability. What remains unexplained is what tips a locus from one regime to the other. Because both regimes rely on the same core machinery—Pol II, Mediator, and their associated IDRs—the determinant is unlikely to lie there, and more likely lies in the valency, concentration, and binding‐site architecture of the gene‐specific layer that recruits and modulates this core machinery. Establishing how that layer sets a locus's position on the spectrum is therefore the more consequential question, rather than deciding whether any individual punctum crosses a phase boundary.

The deeper obstacle to studying this middle ground is that the interactions building these compartments cannot be removed without removing the molecule that carries them, and a single IDR rarely does just one thing. Deleting or mutating an IDR abolishes condensation and the underlying multivalent contacts at once, but the same region typically also mediates defined, structurally characterized interactions with downstream factors, such as the activation‐domain contacts that recruit Mediator. A loss of transcriptional output, therefore, cannot be assigned to loss of the compartment rather than to loss of a specific recruitment function or of the multivalent interactions themselves; the perturbation rarely isolates the variable of interest. This confound runs through nearly every functional claim in the field and is a major reason so much evidence stays correlative. Breaking it requires perturbations that separate condensation from the interactions that drive it, and those interactions from one another, the single methodological advance that would do most to settle the questions above.

We believe that real progress in this field will require novel interdisciplinary approaches that holistically integrate protein biochemistry, structural biophysics, quantitative cell biological imaging, biophysical measurements, mesoscopic simulations, and theoretical approaches from biological physics. Studying the function of individual proteins in buffer one by one has given the community a great deal of insights into transcription compartmentalization, including functional enzymatic properties and detailed structural information. We argue that the information encoded by the “community” of proteins that exist within the compartment is also essential. This argues for increasingly complicated in vitro reconstitution‐based approaches, with more and more factors being correctly added at the appropriate stoichiometry. But reconstitution is a means, not an end. A defined system is where the rules can be isolated, yet function is a cellular property, and the rules it reveals stay hypothetical until they are carried up through more complex settings, from lysates to the intact nucleus, and shown to reproduce function there.

Even then, no current method, in vitro or in cell, yet captures everything that matters. Composition, structure, and dynamics can each be measured, but not together at the scale these compartments occupy, and it is this gap that new methods must close. Emerging structural methods illustrate the kind of advance that will be needed. Most structural techniques resolve a complex by averaging many copies of it, which requires assuming they share a single conformation—an assumption that breaks down for assemblies as heterogeneous and dynamic as transcriptional compartments. Newer single‐molecule approaches instead classify the individual particles in a tomography or electron‐microscopy dataset by shape, allowing several coexisting conformations of the same protein to be reconstructed rather than averaged away [125]. This begins to capture the conformational heterogeneity these compartments depend on, but such methods are still in their early days and remain blind to fast dynamics. Closing that gap will require new and inventive methods, and developing them is what will determine how far we can understand these compartments.

The methodological gap described above represents one example among a wider set of open issues in this field. Below, we summarize what we consider to be the most important of these remaining challenges and open questions:

1. **Stoichiometry and transcriptional output**. How can we quantitatively measure the stoichiometry of transcriptional assemblies in living cells, and how does molecular composition relate to gene activation? Defining stoichiometries by using metrics such as molecular footprints would provide well‐defined units for understanding assembly function. More broadly, transcriptional assemblies may exist along a continuum from oligomeric complexes to large condensates; determining where functionally relevant assemblies fall on this spectrum remains an open challenge.
2. **Necessity versus sufficiency**. Are transcriptional assemblies necessary for robust gene expression, or do they emerge as a consequence of high local concentrations without contributing directly to transcriptional output? If they are functional, what specific roles do they serve—selective recruitment of factors, local concentration of the transcriptional machinery, or spatial segregation of components within the complex nuclear environment? Distinguishing between these possibilities requires perturbations orthogonal to canonical regulation: removing the multivalent IDR contacts that drive condensation while sparing specific cofactor interactions, acutely dissolving or inducing compartments with optogenetic or degron tools, or forcing condensation with engineered scaffolds to test sufficiency, each read out against nascent transcription. The same multivalent contacts, however, often drive condensation and the specific interactions canonical regulation depends on simultaneously, making the two hard to cleanly separate.
3. **Binding specificity**. How do IDR‐mediated interactions achieve specificity in the absence of defined binding interfaces? TFs recognize DNA through well‐characterized sequence motifs, but we lack a predictive framework for specificity encoded in disordered regions. This challenge is compounded by the experimental difficulty of characterizing interactions between IDRs that remain disordered upon binding, as most structural methods rely on stable conformations. While NMR and smFRET have provided valuable insights into such dynamic complexes, these approaches remain limited in throughput compared to methods available for studying DNA‐protein specificity.
4. **Evolutionary persistence of disorder**. Why has evolution maintained IDRs in TFs despite their apparent vulnerability to mutation and aggregation? Eukaryotic TFs are among the most disordered proteins in the proteome, and their IDRs frequently encode transactivation domains essential for transcription and promote interactions required for assembly formation. What selective pressures preserve these regions, and what functional advantages does disorder confer that structured domains cannot provide?
5. **Composition**. What is the full molecular composition of transcriptional assemblies? Current imaging approaches can simultaneously track only a limited number of molecular species, leaving the complete inventory of transcriptional compartments unknown. Fundamental questions remain: How many different molecules occupy a typical transcriptional assembly? Which components are present in what stoichiometry?
6. **Physical mechanism**. Which physical process — LLPS [92], surface condensation [40], or percolation [167] — best describes the formation of transcriptional assemblies in vivo? Each is expected to leave different signatures, in assembly dynamics and reversibility or in whether formation shows a sharp concentration threshold. Distinguishing them inside cells, however, has proven very difficult, and for most assemblies the mechanism remains unresolved.
7. **Generality across systems**. How broadly do the reported mechanisms apply? Sub‐saturation clustering has been characterized for only a handful of proteins, and DNA‐mediated condensation below the bulk saturation concentration has been shown for several pioneer TFs, but whether either extends to non‐pioneer or all phase‐separating TFs is unknown. The functional consequences also vary, with activating, neutral, and inhibitory effects reported across systems, so that generalisation remains difficult. Establishing which principles hold beyond individual model systems will require comparative approaches that test mechanisms systematically across diverse protein families.
8. **Standards and reproducibility**. The field lacks standardization, which limits comparison across laboratories. This spans how assays are run (buffer conditions, crowding agents, full‐length protein versus IDR, temperature reporting), how puncta are interpreted as phase‐separated without establishing material properties or mechanism, how in vitro conditions translate to cellular ones, and inconsistent terminology for both condensates and sub‐saturation clusters. Efforts to catalogue phase‐separation data already exist, such as PhaSepDB, DrLLPS, and CD‐CODE. A more comprehensive and uniformly adopted resource, recording experiments with their exact conditions, as established for model organisms (e.g. WormBase) or structural data (e.g. PDB), would ease comparison and support predictive models of condensation.

## Author Contributions

**T.Q**. and **S.W**.: Conceptualization, Writing – original draft, Writing – review & editing, Visualization, Funding acquisition.

## Conflicts of Interest

The authors declare no conflicts of interest.

## Data Availability

Data sharing not applicable to this article as no datasets were generated or analysed during the current study.

## References

[1] S. A. Lambert , A. Jolma , L. F. Campitelli , et al., “The Human Transcription Factors,” Cell 172, no. 4 (2018): 650–665. 29425488 10.1016/j.cell.2018.01.029 PMC12908702

[2] P. Ding , Y. Wang , X. Zhang , X. Gao , G. Liu , and B. Yu , “DeepSTF: Predicting Transcription Factor Binding Sites by Interpretable Deep Neural Networks Combining Sequence and Shape,” Briefings in Bioinformatics 24, no. 4 (2023): bbad231. 37328639 10.1093/bib/bbad231

[3] S. Schoenfelder and P. Fraser , “Long‐Range Enhancer–Promoter Contacts in Gene Expression Control,” Nature Reviews Genetics 20, no. 8 (2019): 437–455. 10.1038/s41576-019-0128-0 31086298

[4] F. Spitz and E. E. M. Furlong , “Transcription Factors: From Enhancer Binding to Developmental Control,” Nature Reviews Genetics 13, no. 9 (2012): 613–626. 10.1038/nrg3207 22868264

[5] E. E. M. Furlong and M. Levine , “Developmental Enhancers and Chromosome Topology,” Science 361, no. 6409 (2018): 1341–1345. 30262496 10.1126/science.aau0320 PMC6986801

[6] T. Mahmoudi , K. R. Katsani , and C. P. Verrijzer , “GAGA Can Mediate Enhancer Function in Trans by Linking Two Separate DNA Molecules,” The EMBO Journal 21, no. 7 (2002): 1775–1781. 11927561 10.1093/emboj/21.7.1775 PMC125945

[7] A. S. Weintraub , C. H. Li , A. V. Zamudio , et al., “YY1 Is a Structural Regulator of Enhancer‐Promoter Loops,” Cell 171, no. 7 (2017): 1573–1588.e28. 29224777 10.1016/j.cell.2017.11.008 PMC5785279

[8] A. J. Cross , C. M. Jeffries , J. Trewhella , and J. M. L. I. M. Matthews , “LIM Domain Binding Proteins 1 and 2 Have Different Oligomeric States,” Journal of Molecular Biology 399, no. 1 (2010): 133–144. 20382157 10.1016/j.jmb.2010.04.006

[9] N. Petrenko , Y. Jin , K. H. Wong , and K. Struhl , “Mediator Undergoes a Compositional Change During Transcriptional Activation,” Molecular Cell 64, no. 3 (2016): 443–454. 27773675 10.1016/j.molcel.2016.09.015 PMC5096951

[10] L. El Khattabi , H. Zhao , J. Kalchschmidt , et al., “A Pliable Mediator Acts as a Functional Rather Than an Architectural Bridge Between Promoters and Enhancers,” Cell 178 (2019): 1145–1158.e20. 31402173 10.1016/j.cell.2019.07.011 PMC7533040

[11] G. Barshad and C. G. Danko , “Revisiting Models of Enhancer–Promoter Communication in Gene Regulation,” Genome Research 35, no. 6 (2025): 1277–1286. 40456606 10.1101/gr.278389.123 PMC12129020

[12] T. M. Popay and J. R. Dixon , “Coming Full Circle: on the Origin and Evolution of the Looping Model for Enhancer–Promoter Communication,” Journal of Biological Chemistry 298, no. 8 (2022): 102117. 35691341 10.1016/j.jbc.2022.102117 PMC9283939

[13] Y. Kim , Z. Shi , H. Zhang , I. J. Finkelstein , and H. Yu , “Human Cohesin Compacts DNA by Loop Extrusion,” Science 366, no. 6471 (2019): 1345–1349. 31780627 10.1126/science.aaz4475 PMC7387118

[14] S. Golfier , T. Quail , H. Kimura , and J. Brugués , “Cohesin and Condensin Extrude DNA Loops in a Cell Cycle‐Dependent Manner,” Elife 9 (2020): 53885. 10.7554/eLife.53885 PMC7316503 32396063

[15] I. F. Davidson , B. Bauer , D. Goetz , W. Tang , G. Wutz , and J.‐M. Peters , “DNA Loop Extrusion by Human Cohesin,” Science 366, no. 6471 (2019): 1338–1345. 31753851 10.1126/science.aaz3418

[16] Y. Li , J. H. I. Haarhuis , Á. Sedeño Cacciatore , et al., “The Structural Basis for Cohesin–CTCF‐Anchored Loops,” Nature 578, no. 7795 (2020): 472–476. 31905366 10.1038/s41586-019-1910-z PMC7035113

[17] A. J. Faure , D. Schmidt , S. Watt , et al., “Cohesin Regulates Tissue‐Specific Expression by Stabilizing Highly Occupied Cis‐Regulatory Modules,” Genome Research 22, no. 11 (2012): 2163–2175. 22780989 10.1101/gr.136507.111 PMC3483546

[18] M. H. Kagey , J. J. Newman , S. Bilodeau , et al., “Mediator and Cohesin Connect Gene Expression and Chromatin Architecture,” Nature 467, no. 7314 (2010): 430–435. 20720539 10.1038/nature09380 PMC2953795

[19] E. J. Banigan , W. Tang , A. A. van den Berg , et al., “Transcription Shapes 3D Chromatin Organization by Interacting With Loop Extrusion,” Proceedings of the National Academy of Sciences 120, no. 11 (2023): 2210480120. 10.1073/pnas.2210480120 PMC10089175 36897969

[20] S. Ramasamy , A. Aljahani , M. A. Karpinska , et al., “The Mediator Complex Regulates Enhancer‐Promoter Interactions,” Nature Structural & Molecular Biology 30, no. 7 (2023): 991–1000. 10.1038/s41594-023-01027-2 PMC10352134 37430065

[21] P. R. Cook , “The Organization of Replication and Transcription,” Science 284, no. 5421 (1999): 1790–1795. 10364545 10.1126/science.284.5421.1790

[22] B. Tolhuis , R.‐J. Palstra , E. Splinter , F. Grosveld , and W. de Laat , “Looping and Interaction Between Hypersensitive Sites in the Active β‐Globin Locus,” Molecular Cell 10, no. 6 (2002): 1453–1465. 12504019 10.1016/s1097-2765(02)00781-5

[23] K. Kuznetsova , N. M. Chabot , M. Ugolini , et al., “Nanog Organizes Transcription Bodies,” Current Biology 33, no. 1 (2023): 164–173.e5. 36476751 10.1016/j.cub.2022.11.015

[24] F. J. Iborra , A. Pombo , D. A. Jackson , and P. R. Cook , “Active RNA Polymerases Are Localized Within Discrete Transcription ‘Factories’ in human Nuclei,” Journal of Cell Science 109, no. 6 (1996): 1427–1436. 8799830 10.1242/jcs.109.6.1427

[25] C. S. Osborne , L. Chakalova , K. E. Brown , et al., “Active Genes Dynamically Colocalize to Shared Sites of Ongoing Transcription,” Nature Genetics 36, no. 10 (2004): 1065–1071. 15361872 10.1038/ng1423

[26] K. Rippe and A. Papantonis , “RNA Polymerase II Transcription Compartments — From Factories to Condensates,” Nature Reviews Genetics 26, no. 11 (2025): 775–788. 10.1038/s41576-025-00859-6 40537661

[27] B. R. Sabari , A. Dall'Agnese , A. Boija , et al., “Coactivator Condensation at Super‐Enhancers Links Phase Separation and Gene Control,” Science 361, no. 6400 (2018): aar3958. 10.1126/science.aar3958 PMC6092193 29930091

[28] W.‐K. Cho , J.‐H. Spille , M. Hecht , et al., “Mediator and RNA Polymerase II Clusters Associate in Transcription‐Dependent Condensates,” Science 361, no. 6400 (2018): 412–415. 29930094 10.1126/science.aar4199 PMC6543815

[29] I. I. Cisse , I. Izeddin , S. Z. Causse , et al., “Real‐Time Dynamics of RNA Polymerase II Clustering in Live Human Cells,” Science 341, no. 6146 (2013): 664–667. 23828889 10.1126/science.1239053

[30] J. Zuin , G. Roth , Y. Zhan , et al., “Nonlinear Control of Transcription Through Enhancer–Promoter Interactions,” Nature 604, no. 7906 (2022): 571–577. 35418676 10.1038/s41586-022-04570-y PMC9021019

[31] M. Du , S. H. Stitzinger , J.‐H. Spille , et al., “Direct Observation of a Condensate Effect on Super‐Enhancer Controlled Gene Bursting,” Cell 187, no. 2 (2024): 331–344.e17. 38194964 10.1016/j.cell.2023.12.005

[32] S. J. Nair , L. Yang , D. Meluzzi , et al., “Phase Separation of Ligand‐Activated Enhancers Licenses Cooperative Chromosomal Enhancer Assembly,” Nature Structural & Molecular Biology 26, no. 3 (2019): 193–203. 10.1038/s41594-019-0190-5 PMC6709854 30833784

[33] A. Boija , I. A. Klein , B. R. Sabari , et al., “Transcription Factors Activate Genes Through the Phase‐Separation Capacity of Their Activation Domains,” Cell 175, no. 7 (2018): 1842–1855.e16. 30449618 10.1016/j.cell.2018.10.042 PMC6295254

[34] M. Boehning , C. Dugast‐Darzacq , M. Rankovic , et al., “RNA Polymerase II Clustering Through Carboxy‐Terminal Domain Phase Separation,” Nature Structural & Molecular Biology 25, no. 9 (2018): 833–840. 10.1038/s41594-018-0112-y 30127355

[35] M. Dejosez , A. Dall'Agnese , M. Ramamoorthy , et al., “Regulatory Architecture of Housekeeping Genes Is Driven by Promoter Assemblies,” Cell Reports 42, no. 5 (2023): 112505. 37182209 10.1016/j.celrep.2023.112505 PMC10329844

[36] M. J. Lercher , A. O. Urrutia , and L. D. Hurst , “Clustering of Housekeeping Genes Provides a Unified Model of Gene Order in the Human Genome,” Nature Genetics 31, no. 2 (2002): 180–183. 11992122 10.1038/ng887

[37] H.‐W. Nützmann , C. Scazzocchio , and A. Osbourn , “Metabolic Gene Clusters in Eukaryotes,” Annual Review of Genetics 52, no. 1 (2018): 159–183. 10.1146/annurev-genet-120417-031237 30183405

[38] M. Corrales , A. Rosado , R. Cortini , J. van Arensbergen , B. van Steensel , and G. J. Filion , “Clustering of Drosophila Housekeeping Promoters Facilitates Their Expression,” Genome Research 27, no. 7 (2017): 1153–1161. 28420691 10.1101/gr.211433.116 PMC5495067

[39] A. A. Hyman , C. A. Weber , and F. Jülicher , “Liquid‐Liquid Phase Separation in Biology,” Annual Review of Cell and Developmental Biology 30, no. 1 (2014): 39–58. 10.1146/annurev-cellbio-100913-013325 25288112

[40] F. Jülicher and C. A. Weber , “Droplet Physics and Intracellular Phase Separation,” Annual Review of Condensed Matter Physics 15, no. 1 (2024): 237–261.

[41] S. F. Shimobayashi , P. Ronceray , D. W. Sanders , M. P. Haataja , and C. P. Brangwynne , “Nucleation Landscape of Biomolecular Condensates,” Nature 599, no. 7885 (2021): 503–506. 34552246 10.1038/s41586-021-03905-5

[42] J. A. Morin , S. Wittmann , S. Choubey , et al., “Sequence‐Dependent Surface Condensation of a Pioneer Transcription Factor on DNA,” Nature Physics 18, no. 3 (2022): 271–276.

[43] T. Quail , S. Golfier , M. Elsner , et al., “Force Generation by Protein–DNA Co‐Condensation,” Nature Physics 17, no. 9 (2021): 1007–1012.

[44] B. Gouveia , Y. Kim , J. W. Shaevitz , S. Petry , H. A. Stone , and C. P. Brangwynne , “Capillary Forces Generated by Biomolecular Condensates,” Nature 609, no. 7926 (2022): 255–264. 36071192 10.1038/s41586-022-05138-6

[45] J. W. Cahn , “Critical Point Wetting,” The Journal of Chemical Physics 66, no. 8 (1977): 3667–3672.

[46] X. Zhao , G. Bartolucci , A. Honigmann , F. Jülicher , and C. A. Weber , “Thermodynamics of Wetting, Prewetting and Surface Phase Transitions With Surface Binding,” New Journal of Physics 23, no. 12 (2021): 123003.

[47] T. Nguyen , S. Li , J. T.‐H. Chang , et al., “Chromatin Sequesters Pioneer Transcription Factor Sox2 from Exerting Force on DNA,” Nature Communications 13, no. 1 (2022): 3988. 10.1038/s41467-022-31738-x PMC9271091 35810158

[48] J. Z. Zhao , J. Xia , and C. P. Brangwynne , “Chromatin Compaction During Confined Cell Migration Induces and Reshapes Nuclear Condensates,” Nature Communications 15, no. 1 (2024): 9964. 10.1038/s41467-024-54120-5 PMC11574006 39557835

[49] A. R. Strom , Y. Kim , H. Zhao , et al., “Condensate Interfacial Forces Reposition DNA Loci and Probe Chromatin Viscoelasticity,” Cell 187, no. 19 (2024): 5282–5297.e20. 39168125 10.1016/j.cell.2024.07.034

[50] D. Hnisz , K. Shrinivas , R. A. Young , A. K. Chakraborty , and P. A. Sharp , “A Phase Separation Model for Transcriptional Control,” Cell 169, no. 1 (2017): 13–23. 28340338 10.1016/j.cell.2017.02.007 PMC5432200

[51] A. S. Lyon , W. B. Peeples , and M. K. Rosen , “A Framework for Understanding the Functions of Biomolecular Condensates Across Scales,” Nature Reviews Molecular Cell Biology 22, no. 3 (2021): 215–235. 33169001 10.1038/s41580-020-00303-z PMC8574987

[52] M. Palacio and D. J. Taatjes , “Merging Established Mechanisms With New Insights: Condensates, Hubs, and the Regulation of RNA Polymerase II Transcription,” Journal of Molecular Biology 434, no. 1 (2022): 167216. 34474085 10.1016/j.jmb.2021.167216 PMC8748285

[53] W. Peeples and M. K. Rosen , “Mechanistic Dissection of Increased Enzymatic Rate in a Phase‐separated Compartment,” Nature Chemical Biology 17, no. 6 (2021): 693–702. 34035521 10.1038/s41589-021-00801-x PMC8635274

[54] F. Stoffel , M. Papp , M. Gil‐Garcia , et al., “Enhancement of Enzymatic Activity by Biomolecular Condensates Through pH Buffering,” Nature Communications 16, no. 1 (2025): 6368. 10.1038/s41467-025-61013-8 PMC12246476 40640131

[55] W. Y. C. Huang , S. Alvarez , Y. Kondo , et al., “A Molecular Assembly Phase Transition and Kinetic Proofreading Modulate Ras Activation by SOS,” Science 363, no. 6431 (2019): 1098–1103. 30846600 10.1126/science.aau5721 PMC6563836

[56] G. Pei , H. Lyons , P. Li , and B. R. Sabari , “Transcription Regulation by Biomolecular Condensates,” Nature Reviews Molecular Cell Biology 26, no. 3 (2025): 213–236. 39516712 10.1038/s41580-024-00789-x PMC12186837

[57] P. Gan , M. Eppert , N. De La Cruz , et al., “Coactivator Condensation Drives Cardiovascular Cell Lineage Specification,” Science Advances 10, no. 11 (2024): adk7160. 10.1126/sciadv.adk7160 PMC10942106 38489358

[58] Z. Li , L. Luo , W. Yu , et al., “PPARγ Phase Separates With RXRα at PPREs to Regulate Target Gene Expression,” Cell Discovery 8, no. 1 (2022): 37. 35473936 10.1038/s41421-022-00388-0 PMC9043196

[59] H. Zhang , S. Shao , Y. Zeng , et al., “Reversible Phase Separation of HSF1 Is Required for an Acute Transcriptional Response During Heat Shock,” Nature Cell Biology 24, no. 3 (2022): 340–352. 35256776 10.1038/s41556-022-00846-7

[60] S. C. Tang , U. Vijayakumar , Y. Zhang , and M. J. Fullwood , “Super‐Enhancers, Phase‐Separated Condensates, and 3D Genome Organization in Cancer,” Cancers 14, no. 12 (2022): 2866. 35740532 10.3390/cancers14122866 PMC9221043

[61] N. Schneider , F.‐G. Wieland , D. Kong , et al., “Liquid‐Liquid Phase Separation of Light‐Inducible Transcription Factors Increases Transcription Activation in Mammalian Cells and Mice,” Science Advances 7, no. 1 (2021): abd3568. 10.1126/sciadv.abd3568 PMC7775772 33523844

[62] M.‐T. Wei , Y.‐C. Chang , S. F. Shimobayashi , Y. Shin , A. R. Strom , and C. P. Brangwynne , “Nucleated Transcriptional Condensates Amplify Gene Expression,” Nature Cell Biology 22, no. 10 (2020): 1187–1196. 32929202 10.1038/s41556-020-00578-6

[63] J. Trojanowski , L. Frank , A. Rademacher , N. Mücke , P. Grigaitis , and K. Rippe , “Transcription Activation Is Enhanced by Multivalent Interactions Independent of Phase Separation,” Molecular Cell 82, no. 10 (2022): 1878–1893.e10. 35537448 10.1016/j.molcel.2022.04.017

[64] G. Gill and M. Ptashne , “Negative Effect of the Transcriptional Activator GAL4,” Nature 334, no. 6184 (1988): 721–724. 3412449 10.1038/334721a0

[65] M. Mazzocca , A. Loffreda , E. Colombo , et al., “Chromatin Organization Drives the Search Mechanism of Nuclear Factors,” Nature Communications 14, no. 1 (2023): 6433. 10.1038/s41467-023-42133-5 PMC10575952 37833263

[66] J. V. W. Meeussen and T. L. Lenstra , “Time Will Tell: Comparing Timescales to Gain Insight Into Transcriptional Bursting,” Trends in Genetics 40, no. 2 (2024): 160–174. 38216391 10.1016/j.tig.2023.11.003 PMC10860890

[67] N. Galvanetto , M. T. Ivanovic , A. Chowdhury , et al., “Extreme Dynamics in a Biomolecular Condensate,” Nature 619, no. 7971 (2023): 876–883. 37468629 10.1038/s41586-023-06329-5 PMC11508043

[68] C.‐Y. Cho and P. H. O'Farrell , “Stepwise Modifications of Transcriptional Hubs Link Pioneer Factor Activity to a Burst of Transcription,” Nature Communications 14, no. 1 (2023): 4848. 10.1038/s41467-023-40485-6 PMC10415302 37563108

[69] W.‐K. Cho , N. Jayanth , B. P. English , et al., “RNA Polymerase II Cluster Dynamics Predict mRNA Output in Living Cells,” eLife 5 (2016): 13617. 10.7554/eLife.13617 PMC4929003 27138339

[70] F. J. DeHaro‐Arbona , C. Roussos , S. Baloul , J. Townson , M. J. Gómez Lamarca , and S. Bray , “Dynamic Modes of Notch Transcription Hubs Conferring Memory and Stochastic Activation Revealed by Live Imaging the Co‐Activator Mastermind,” eLife 12 (2024): RP92083. 38727722 10.7554/eLife.92083 PMC11087053

[71] S. Wang , T. Suter , A. Gamliel , et al., “Endogenous Real Time Imaging Reveals Dynamic Chromosomal Mobility During Ligand‐Mediated Transcriptional Burst Events,” eLife 14 (2025): RP108726.

[72] M. Levo , J. Raimundo , X. Y. Bing , et al., “Transcriptional Coupling of Distant Regulatory Genes in Living Embryos,” Nature 605, no. 7911 (2022): 754–760. 35508662 10.1038/s41586-022-04680-7 PMC9886134

[73] J. V. W. Meeussen , W. Pomp , I. Brouwer , W. J. de Jonge , H. P. Patel , and T. L. Lenstra , “Transcription Factor Clusters Enable Target Search but Do Not Contribute to Target Gene Activation,” Nucleic Acids Research 51, no. 11 (2023): 5449–5468. 36987884 10.1093/nar/gkad227 PMC10287935

[74] W. Pomp , J. V. W. Meeussen , and T. L. Lenstra , “Transcription Factor Exchange Enables Prolonged Transcriptional Bursts,” Molecular Cell 84, no. 6 (2024): 1036–1048.e9. 38377994 10.1016/j.molcel.2024.01.020 PMC10962226

[75] F. Erdel and K. Rippe , “Formation of Chromatin Subcompartments by Phase Separation,” Biophysical Journal 114, no. 10 (2018): 2262–2270. 29628210 10.1016/j.bpj.2018.03.011 PMC6129460

[76] H. Zhang , W. Qin , H. Romero , H. Leonhardt , and M. C. Cardoso , “Heterochromatin Organization and Phase Separation,” Nucleus 14, no. 1 (2023): 2159142. 36710442 10.1080/19491034.2022.2159142 PMC9891170

[77] N. Treen , S. F. Shimobayashi , J. Eeftens , C. P. Brangwynne , and M. Levine , “Properties of Repression Condensates in Living Ciona Embryos,” Nature Communications 12, no. 1 (2021): 1561. 10.1038/s41467-021-21606-5 PMC7946874 33692345

[78] Y. Yamaguchi , T. Takagi , T. Wada , et al., “NELF, a Multisubunit Complex Containing RD, Cooperates With DSIF to Repress RNA Polymerase II Elongation,” Cell 97, no. 1 (1999): 41–51. 10199401 10.1016/s0092-8674(00)80713-8

[79] P. Rawat , M. Boehning , B. Hummel , et al., “Stress‐Induced Nuclear Condensation of NELF Drives Transcriptional Downregulation,” Molecular Cell 81, no. 5 (2021): 1013–1026.e11. 33548202 10.1016/j.molcel.2021.01.016 PMC7939545

[80] S. Jiang , Z. Jia , W. Zhu , et al., “HSPA1A and DNAJB1 Regulate NELF Condensate Dynamics to Safeguard Transcriptional Recovery Under Heat Stress,” Molecular Cell 86, no. 4 (2026): 674–692.e10. 41653920 10.1016/j.molcel.2026.01.015

[81] S. Zhu , X. Zhang , N. Li , et al., “MED26‐enriched Condensates Drive Erythropoiesis Through Modulating Transcription Pausing,” eLife 13 (2024): RP102023.

[82] C. J. Weaver , A. L. Patel , S. Y. Shvartsman , M. S. Levine , and N. Treen , “ERK Signaling Dissolves ERF Repression Condensates in Living Embryos,” Proceedings of the National Academy of Sciences 119, no. 9 (2022): 2119187119. 10.1073/pnas.2119187119 PMC8892517 35217620

[83] T. N. Medwig‐Kinney , B. A. Kinney , M. A. Q. Martinez , et al., “Dynamic Compartmentalization of the Pro‐Invasive Transcription Factor NHR‐67 Reveals a Role for Groucho in Regulating a Proliferative‐Invasive Cellular Switch in C. elegans,” eLife 12 (2023): RP84355. 38038410 10.7554/eLife.84355 PMC10691804

[84] Y. Huang , Z. Liang , and P. Xia , “Liquid–Liquid Phase Separation of Transcription Factors Facilitates Environmental Adaptation in Plants,” Plant Physiology 199 (2025): kiaf377. 40878008 10.1093/plphys/kiaf377

[85] S. Hutin , J. R. Kumita , V. I. Strotmann , et al., “Phase Separation and Molecular Ordering of the Prion‐Like Domain of the Arabidopsis Thermosensory Protein EARLY FLOWERING 3,” Proceedings of the National Academy of Sciences 120, no. 28 (2023): 2304714120. 10.1073/pnas.2304714120 PMC10334799 37399408

[86] P. Zhu , C. Lister , and C. Dean , “Cold‐induced Arabidopsis FRIGIDA Nuclear Condensates for FLC Repression,” Nature 599, no. 7886 (2021): 657–661. 34732891 10.1038/s41586-021-04062-5 PMC8612926

[87] D. Cai , D. Feliciano , P. Dong , et al., “Phase Separation of YAP Reorganizes Genome Topology for Long‐term YAP Target Gene Expression,” Nature Cell Biology 21, no. 12 (2019): 1578–1589. 31792379 10.1038/s41556-019-0433-z PMC8259329

[88] Y. Tang , F. Chen , G. Fang , et al., “A Cofactor‐Induced Repressive Type of Transcription Factor Condensation Can be Induced by Synthetic Peptides to Suppress Tumorigenesis,” The EMBO Journal 43, no. 22 (2024): 5586–5612. 39358623 10.1038/s44318-024-00257-4 PMC11574045

[89] B. R. Sabari , A. Dall'Agnese , and R. A. Young , “Biomolecular Condensates in the Nucleus,” Trends in Biochemical Sciences 45, no. 11 (2020): 961–977. 32684431 10.1016/j.tibs.2020.06.007 PMC7572565

[90] M. R. King , K. M. Ruff , A. Z. Lin , et al., “Macromolecular Condensation Organizes Nucleolar Sub‐Phases to Set Up a pH Gradient,” Cell 187, no. 8 (2024): 1889–1906.e24. 38503281 10.1016/j.cell.2024.02.029 PMC11938373

[91] H. Ausserwöger , R. Scrutton , C. M. Fischer , et al., “Biomolecular Condensates Sustain pH Gradients at Equilibrium through Charge Neutralization,” Nature Chemistry 18, no. 2 (2026): 246–257. 10.1038/s41557-025-02039-9 PMC12872462 41612036

[92] S. F. Banani , H. O. Lee , A. A. Hyman , and M. K. Rosen , “Biomolecular Condensates: Organizers of Cellular Biochemistry,” Nature Reviews Molecular Cell Biology 18, no. 5 (2017): 285–298. 28225081 10.1038/nrm.2017.7 PMC7434221

[93] A. S. Holehouse and B. B. Kragelund , “The Molecular Basis for Cellular Function of Intrinsically Disordered Protein Regions,” Nature Reviews Molecular Cell Biology 25, no. 3 (2024): 187–211. 37957331 10.1038/s41580-023-00673-0 PMC11459374

[94] Y. E. Guo , J. C. Manteiga , J. E. Henninger , et al., “Pol II Phosphorylation Regulates a Switch Between Transcriptional and Splicing Condensates,” Nature 572, no. 7770 (2019): 543–548. 31391587 10.1038/s41586-019-1464-0 PMC6706314

[95] H. Lyons , R. T. Veettil , P. Pradhan , et al., “Functional Partitioning of Transcriptional Regulators by Patterned Charge Blocks,” Cell 186, no. 2 (2023): 327–345.e28. 36603581 10.1016/j.cell.2022.12.013 PMC9910284

[96] M. D. Driver and P. R. Onck , “Selective Phase Separation of Transcription Factors Is Driven by Orthogonal Molecular Grammar,” Nature Communications 16, no. 1 (2025): 3087. 10.1038/s41467-025-58445-7 PMC11958648 40164612

[97] S. Chong , C. Dugast‐Darzacq , Z. Liu , et al., “Imaging Dynamic and Selective Low‐Complexity Domain Interactions That Control Gene Transcription,” Science 361, no. 6400 (2018): aar2555. 10.1126/science.aar2555 PMC6961784 29930090

[98] D. T. McSwiggen , A. S. Hansen , S. S. Teves , et al., “Evidence for DNA‐Mediated Nuclear Compartmentalization Distinct From Phase Separation,” Elife 8 (2019): 47098. 10.7554/eLife.47098 PMC6522219 31038454

[99] S. Basu , S. D. Mackowiak , H. Niskanen , et al., “Unblending of Transcriptional Condensates in Human Repeat Expansion Disease,” Cell 181, no. 5 (2020): 1062–1079.e30. 32386547 10.1016/j.cell.2020.04.018 PMC7261253

[100] M. A. Mensah , H. Niskanen , A. P. Magalhaes , et al., “Aberrant Phase Separation and Nucleolar Dysfunction in Rare Genetic Diseases,” Nature 614, no. 7948 (2023): 564–571. 36755093 10.1038/s41586-022-05682-1 PMC9931588

[101] K. Wagh , D. A. Stavreva , and G. L. Hager , “Transcription Dynamics and Genome Organization in the Mammalian Nucleus: Recent Advances,” Molecular Cell 85, no. 2 (2025): 208–224. 39413793 10.1016/j.molcel.2024.09.022 PMC11741928

[102] Z. Xie , I. Sokolov , M. Osmala , et al., “DNA‐Guided Transcription Factor Interactions Extend Human Gene Regulatory Code,” Nature 641, no. 8065 (2025): 1329–1338. 40205063 10.1038/s41586-025-08844-z PMC12119339

[103] J. M. Schaepe , T. Fries , B. R. Doughty , et al., “Thermodynamic Principles Link in Vitro Transcription Factor Affinities to Single‐Molecule Chromatin States in Cells,” Cell 189, no. 1 (2026): 307–322.e23. 41308636 10.1016/j.cell.2025.11.008 PMC13397507

[104] O. G. Berg , R. B. Winter , and P. H. Von Hippel , “Diffusion‐Driven Mechanisms of Protein Translocation on Nucleic Acids. 1. Models and Theory,” Biochemistry 20, no. 24 (1981): 6929–6948. 7317363 10.1021/bi00527a028

[105] L. Mirny , M. Slutsky , Z. Wunderlich , A. Tafvizi , J. Leith , and A. Kosmrlj , “How a Protein Searches for Its Site on DNA: The Mechanism of Facilitated Diffusion,” Journal of Physics A: Mathematical and Theoretical 42, no. 43 (2009): 434013.

[106] A. Tafvizi , F. Huang , J. S. Leith , A. R. Fersht , L. A. Mirny , and A. M. van Oijen , “Tumor Suppressor p53 Slides on DNA With Low Friction and High Stability,” Biophysical Journal 95, no. 1 (2008): L01–L03. 18424488 10.1529/biophysj.108.134122 PMC2426630

[107] X. A. Feng , M. Yamadi , Y. Fu , et al., “GAGA Zinc Finger Transcription Factor Searches Chromatin by 1D–3D Facilitated Diffusion,” Nature Structural & Molecular Biology 32, no. 11 (2025): 2359–2370. 10.1038/s41594-025-01643-0 PMC12618267 40764461

[108] E. Marklund , G. Mao , J. Yuan , et al., “Sequence Specificity in DNA Binding Is Mainly Governed by Association,” Science 375, no. 6579 (2022): 442–445. 35084952 10.1126/science.abg7427

[109] S. Inukai , K. H. Kock , and M. L. Bulyk , “Transcription Factor–DNA Binding: Beyond Binding Site Motifs,” Current Opinion in Genetics & Development 43 (2017): 110–119. 28359978 10.1016/j.gde.2017.02.007 PMC5447501

[110] L. Isbel , R. S. Grand , and D. Schübeler , “Generating Specificity in Genome Regulation Through Transcription Factor Sensitivity to Chromatin,” Nature Reviews Genetics 23, no. 12 (2022): 728–740. 10.1038/s41576-022-00512-6 35831531

[111] A. A. Abidi , C. Cattoglio , N. N. Tang , et al., “Unstructured Transcription Factor Interactions Enable Emergent Specificity,” Science 392, no. 6801 (2026): aeb6487.10.1126/science.aeb648741855276

[112] G. Noviello , “Transcriptional Regulation as a Dose‐Dependent Process: Insights From Transcription Factor Tuning,” Open Biology 15, no. 8 (2025): 240328. 40763802 10.1098/rsob.240328 PMC12324873

[113] P. Cramer , “Eukaryotic Transcription Turns 50,” Cell 179, no. 4 (2019): 808–812. 31675494 10.1016/j.cell.2019.09.018

[114] A. Lobley , M. B. Swindells , C. A. Orengo , and D. T. Jones , “Inferring Function Using Patterns of Native Disorder in Proteins,” PLoS Computational Biology 3, no. 8 (2007): 162. 10.1371/journal.pcbi.0030162 PMC1950950 17722973

[115] K. Cermakova and H. C. Hodges , “Interaction Modules That Impart Specificity to Disordered Protein,” Trends in Biochemical Sciences 48, no. 5 (2023): 477–490. 36754681 10.1016/j.tibs.2023.01.004 PMC10106370

[116] H. K. Shirnekhi , B. Chandra , and R. W. Kriwacki , “The Role of Phase‐Separated Condensates in Fusion Oncoprotein–Driven Cancers,” Annual Review of Cancer Biology 7, no. 1 (2023): 73–91. 10.1146/annurev-cancerbio-061421-122050 PMC13193247 42180996

[117] X. Q. D. Wang , D. Fan , Q. Han , et al., “Mutant NPM1 Hijacks Transcriptional Hubs to Maintain Pathogenic Gene Programs in Acute Myeloid Leukemia,” Cancer Discovery 13, no. 3 (2023): 724–745. 36455589 10.1158/2159-8290.CD-22-0424 PMC9975662

[118] L. Song , X. Yao , H. Li , et al., “Hotspot Mutations in the Structured ENL YEATS Domain Link Aberrant Transcriptional Condensates and Cancer,” Molecular Cell 82, no. 21 (2022): 4080–4098.e12. 36272410 10.1016/j.molcel.2022.09.034 PMC10071517

[119] I. Yruela , C. J. Oldfield , K. J. Niklas , and A. K. Dunker , “Evidence for a Strong Correlation between Transcription Factor Protein Disorder and Organismic Complexity,” Genome Biology and Evolution 9, no. 5 (2017): 1248–1265. 28430951 10.1093/gbe/evx073 PMC5434936

[120] M. Már , K. Nitsenko , and P. O. Heidarsson , “Multifunctional Intrinsically Disordered Regions in Transcription Factors,” Chemistry–A European Journal 29 (2023): 202203369. 10.1002/chem.202203369 36648282

[121] S. Piskacek , M. Gregor , M. Nemethova , M. Grabner , P. Kovarik , and M. Piskacek , “Nine‐Amino‐Acid Transactivation Domain: Establishment and Prediction Utilities,” Genomics 89, no. 6 (2007): 756–768. 17467953 10.1016/j.ygeno.2007.02.003

[122] T. Zor , R. N. De Guzman , H. J. Dyson , and P. E. Wright , “Solution Structure of the KIX Domain of CBP Bound to the Transactivation Domain of c‐Myb,” Journal of Molecular Biology 337, no. 3 (2004): 521–534. 15019774 10.1016/j.jmb.2004.01.038

[123] I. Radhakrishnan , G. C. Pérez‐Alvarado , D. Parker , H. J. Dyson , M. R. Montminy , and P. E. Wright , “Solution Structure of the KIX Domain of CBP Bound to the Transactivation Domain of CREB: A Model for Activator: Coactivator Interactions,” Cell 91, no. 6 (1997): 741–752. 9413984 10.1016/s0092-8674(00)80463-8

[124] R. Marmorstein and M.‐M. Zhou , “Writers and Readers of Histone Acetylation: Structure, Mechanism, and Inhibition,” Cold Spring Harbor Perspectives in Biology 6, no. 7 (2014): a018762–a018762. 24984779 10.1101/cshperspect.a018762 PMC4067988

[125] P. S. Brzovic , C. C. Heikaus , L. Kisselev , et al., “The Acidic Transcription Activator Gcn4 Binds the Mediator Subunit Gal11/Med15 Using a Simple Protein Interface Forming a Fuzzy Complex,” Molecular Cell 44, no. 6 (2011): 942–953. 22195967 10.1016/j.molcel.2011.11.008 PMC3246216

[126] L. M. Tuttle , D. Pacheco , L. Warfield , et al., “Gcn4‐Mediator Specificity Is Mediated by a Large and Dynamic Fuzzy Protein‐Protein Complex,” Cell Reports 22, no. 12 (2018): 3251–3264. 29562181 10.1016/j.celrep.2018.02.097 PMC5908246

[127] L. M. Tuttle , D. Pacheco , L. Warfield , D. B. Wilburn , S. Hahn , and R. E. Klevit , “Mediator Subunit Med15 Dictates the Conserved “Fuzzy” Binding Mechanism of Yeast Transcription Activators Gal4 and Gcn4,” Nature Communications 12, no. 1 (2021): 2220. 10.1038/s41467-021-22441-4 PMC8044209 33850123

[128] V. Csizmok and J. D. Forman‐Kay , “Complex Regulatory Mechanisms Mediated by the Interplay of Multiple Post‐Translational Modifications,” Current Opinion in Structural Biology 48 (2018): 58–67. 29100108 10.1016/j.sbi.2017.10.013

[129] A. C. Murthy , W. S. Tang , N. Jovic , et al., “Molecular Interactions Contributing to FUS SYGQ LC‐RGG Phase Separation and Co‐Partitioning with RNA Polymerase II Heptads,” Nature Structural & Molecular Biology 28, no. 11 (2021): 923–935. 10.1038/s41594-021-00677-4 PMC8654040 34759379

[130] J. A. Riback , L. Zhu , M. C. Ferrolino , et al., “Composition‐Dependent Thermodynamics of Intracellular Phase Separation,” Nature 581, no. 7807 (2020): 209–214. 32405004 10.1038/s41586-020-2256-2 PMC7733533

[131] N. M. Milkovic and T. Mittag , “Determination of Protein Phase Diagrams by Centrifugation,” in Intrinsically Disordered Proteins: Methods and Protocols (Springer, 2020), 685–702. 10.1007/978-1-0716-0524-0_35 32696384

[132] A. W. Fritsch , J. M. Iglesias‐Artola , and A. A. Hyman , “inPhase — A Simple, Accurate and Fast Approach to Determine Phase Diagrams of Protein Condensates,” bioRxiv (2024), 10.1101/2024.10.02.616352.

[133] W. E. Arter , R. Qi , N. A. Erkamp , et al., “Biomolecular Condensate Phase Diagrams With a Combinatorial Microdroplet Platform,” Nature Communications 13, no. 1 (2022): 7845. 10.1038/s41467-022-35265-7 PMC9768726 36543777

[134] S. Ray and A. K. Buell , “Emerging Experimental Methods to Study the Thermodynamics of Biomolecular Condensate Formation,” The Journal of Chemical Physics 160, no. 9 (2024): 091001. 38445729 10.1063/5.0190160

[135] P. M. McCall , K. Kim , A. Shevchenko , et al., “A Label‐Free Method for Measuring the Composition of Multicomponent Biomolecular Condensates,” Nature Chemistry 17, no. 12 (2025): 1891–1902. 10.1038/s41557-025-01928-3 PMC12669041 40903498

[136] K. Hertäg , S. Shoup , L. T. Thews , et al., “Active Field Theory Approach to Explain Size Control of Transcriptional Condensates,” bioRxiv (2026), 10.64898/2026.05.17.725716.

[137] Y. Ge , T. Paul , M. Gordiychuk , N. Das , Y. Zhang , and S. Myong , “FUS Nanoclusters Are a Distinct State Within the Dilute Phase,” Nature Communications 16, no. 1 (2025): 9956. 10.1038/s41467-025-64909-7 PMC12612064 41224766

[138] M. Kar , F. Dar , T. J. Welsh , et al., “Phase‐Separating RNA‐Binding Proteins Form Heterogeneous Distributions of Clusters in Subsaturated Solutions,” Proceedings of the National Academy of Sciences 119, no. 28 (2022): 2202222119. 10.1073/pnas.2202222119 PMC9282234 35787038

[139] M. K. Shinn , D. T. Tomares , V. Liu , et al., “Nuclear Speckle Proteins Form Intrinsic and MALAT1‐Dependent Microphases,” Cell 189, no. 3 (2026): 832–852.e24. 41421357 10.1016/j.cell.2025.11.026 PMC12922802

[140] H. Ruan , R. F. Dillenburg , E. Hosseini , S. Wittmann , M. Girard , and E. A. Lemke , “Differential Conformational Expansion of NUP98‐HOXA9 Oncoprotein from Nanosized Assemblies to Macrophases,” Nature Communications 16, no. 1 (2025): 10117. 10.1038/s41467-025-66327-1 PMC12627784 41253788

[141] M. Mir , A. Reimer , J. E. Haines , et al., “Dense Bicoid Hubs Accentuate Binding Along the Morphogen Gradient,” Genes & Development 31, no. 17 (2017): 1784–1794. 28982761 10.1101/gad.305078.117 PMC5666676

[142] L. Black , S. Tollis , G. Fu , et al., “G1/S Transcription Factors Assemble in Increasing Numbers of Discrete Clusters Through G1 Phase,” Journal of Cell Biology 219, no. 9 (2020): 202003041. 10.1083/jcb.202003041 PMC7480102 32744610

[143] B. T. Donovan , A. Huynh , D. A. Ball , et al., “Live‐Cell Imaging Reveals the Interplay Between Transcription Factors, Nucleosomes, and Bursting,” The EMBO Journal 38, no. 12 (2019): 100809. 10.15252/embj.2018100809 PMC6576174 31101674

[144] K. Kawasaki and T. Fukaya , “Functional Coordination between Transcription Factor Clustering and Gene Activity,” Molecular Cell 83, no. 10 (2023): 1605–1622.e9. 37207625 10.1016/j.molcel.2023.04.018

[145] Z. Li , F. Burgos‐Bravo , K. Xu , et al., “Phase‐Separated NDF− FACT Condensates Facilitate Transcription Elongation on Chromatin,” Nature Cell Biology 27, no. 11 (2025): 1938–1951. 41028835 10.1038/s41556-025-01778-8 PMC12611769

[146] M. Becker , C. Baumann , S. John , et al., “Dynamic Behavior of Transcription Factors on a Natural Promoter in Living Cells,” The EMBO Reports 3, no. 12 (2002): 1188–1194. 12446572 10.1093/embo-reports/kvf244 PMC1308318

[147] M. Wachsmuth , W. Waldeck , and J. Langowski , “Anomalous Diffusion of Fluorescent Probes Inside Living Cell Nuclei Investigated by Spatially‐Resolved Fluorescence Correlation Spectroscopy,” Journal of Molecular Biology 298, no. 4 (2000): 677–689. 10788329 10.1006/jmbi.2000.3692

[148] M. D. White , J. F. Angiolini , Y. D. Alvarez , et al., “Long‐Lived Binding of Sox2 to DNA Predicts Cell Fate in the Four‐Cell Mouse Embryo,” Cell 165, no. 1 (2016): 75–87. 27015308 10.1016/j.cell.2016.02.032

[149] E. Hinde , E. Pandzic , Z. Yang , et al., “Quantifying the Dynamics of the Oligomeric Transcription Factor STAT3 by Pair Correlation of Molecular Brightness,” Nature Communications 7, no. 1 (2016): 11047. 10.1038/ncomms11047 PMC4820838 27009358

[150] M. Stortz , D. M. Presman , L. Bruno , et al., “Mapping the Dynamics of the Glucocorticoid Receptor Within the Nuclear Landscape,” Scientific Reports 7, no. 1 (2017): 6219. 28740156 10.1038/s41598-017-06676-0 PMC5524710

[151] P. Mathur , M. Papp , K. Makasewicz , P. Arosio , A. J. deMello , and S. Stavrakis , “Measuring Concentration and Diffusivity Within Biomolecular Condensates Using Calibration‐Free Scanning Fluorescence Correlation Spectroscopy,” Chemical Science 17, no. 2 (2026): 985–995. 41293562 10.1039/d5sc05592j PMC12641978

[152] K. Wagh , D. A. Stavreva , A. Upadhyaya , and G. L. Hager , “Transcription Factor Dynamics: One Molecule at a Time,” Annual Review of Cell and Developmental Biology 39, no. 1 (2023): 277–305. 10.1146/annurev-cellbio-022823-013847 37540844

[153] J. Chen , Z. Zhang , L. Li , et al., “Single‐Molecule Dynamics of Enhanceosome Assembly in Embryonic Stem Cells,” Cell 156, no. 6 (2014): 1274–1285. 24630727 10.1016/j.cell.2014.01.062 PMC4040518

[154] E. E. Swinstead , T. B. Miranda , V. Paakinaho , et al., “Steroid Receptors Reprogram FoxA1 Occupancy Through Dynamic Chromatin Transitions,” Cell 165, no. 3 (2016): 593–605. 27062924 10.1016/j.cell.2016.02.067 PMC4842147

[155] J. C. M. Gebhardt , D. M. Suter , R. Roy , et al., “Single‐Molecule Imaging of Transcription Factor Binding to DNA in Live Mammalian Cells,” Nature Methods 10, no. 5 (2013): 421–426. 23524394 10.1038/nmeth.2411 PMC3664538

[156] D. A. Garcia , G. Fettweis , D. M. Presman , et al., “Power‐Law Behavior of Transcription Factor Dynamics at the Single‐Molecule Level Implies a Continuum Affinity Model,” Nucleic Acids Research 49, no. 12 (2021): 6605–6620. 33592625 10.1093/nar/gkab072 PMC8266587

[157] Z. Kosar and A. Erbas , “Protein‐DNA Clusters Explain the Non‐Exponential DNA Residence Time Distributions of Transcription Factors,” bioRxiv (2023), 10.1101/2023.09.21.558872.

[158] Y. H. Ling , C. Liang , S. Wang , and C. Wu , “Live‐Cell Single‐Molecule Dynamics of Eukaryotic RNA Polymerase Machineries,” Science 391 (2026): ads0960. 10.1126/science.ads0960 PMC12980801 41642946

[159] A. Castells‐Garcia , I. Ed‐daoui , E. González‐Almela , et al., “Super Resolution Microscopy Reveals How Elongating RNA Polymerase II and Nascent RNA Interact With Nucleosome Clutches,” Nucleic Acids Research 50, no. 1 (2022): 175–190. 34929735 10.1093/nar/gkab1215 PMC8754629

[160] T. Deguchi , M. K. Iwanski , E.‐M. Schentarra , et al., “Direct Observation of Motor Protein Stepping in Living Cells Using MINFLUX,” Science 379, no. 6636 (2023): 1010–1015. 36893247 10.1126/science.ade2676 PMC7614483

[161] M. Mazzocca , D. N. Narducci , S. Grosse‐Holz , and J. Matthias , “Integrated MINFLUX Tracking Reveals Two Distinct Chromatin Dynamics Classes Across Cell Types,” Nature Structural & Molecular Biology 33, no. 6 (2026): 905–914. 10.1038/s41594-026-01807-6 PMC13419685 42082828

[162] B. E. Collins , L. F. Ye , D. Duzdevich , and E. C. Greene , “DNA Curtains: Novel Tools for Imaging Protein–Nucleic Acid Interactions at the Single‐Molecule Level,” in Methods in Cell Biology (Elsevier, 2014), 217–234. 10.1016/B978-0-12-420138-5.00012-4 24974030

[163] M. Ganji , I. A. Shaltiel , S. Bisht , et al., “Real‐Time Imaging of DNA Loop Extrusion by Condensin,” Science 360, no. 6384 (2018): 102–105. 29472443 10.1126/science.aar7831 PMC6329450

[164] C. J. Bustamante , Y. R. Chemla , S. Liu , and M. D. Wang , “Optical Tweezers in Single‐Molecule Biophysics,” Nature Reviews Methods Primers 1, no. 1 (2021): 25. 10.1038/s43586-021-00021-6 PMC8629167 34849486

[165] J. A. Rakowski , M. A. Schaich , B. L. Schnable , and B. Van Houten , “Utilizing Nuclear Extracts to Characterize Protein: DNA Interactions at the Single Molecule Level,” in Methods in Enzymology (Elsevier, 2024), 397–426. 10.1016/bs.mie.2024.07.014 PMC11626518 39389671

[166] Y. Shin and C. P. Brangwynne , “Liquid Phase Condensation in Cell Physiology and Disease,” Science 357, no. 6357 (2017): aaf4382. 10.1126/science.aaf4382 28935776

[167] T. Mittag and R. V. Pappu , “A Conceptual Framework for Understanding Phase Separation and Addressing Open Questions and Challenges,” Molecular Cell 82, no. 12 (2022): 2201–2214. 35675815 10.1016/j.molcel.2022.05.018 PMC9233049

[168] A. L. Gnatt , P. Cramer , J. Fu , D. A. Bushnell , and R. D. Kornberg , “Structural Basis of Transcription: an RNA Polymerase II Elongation Complex at 3.3 Å Resolution,” Science 292, no. 5523 (2001): 1876–1882. 11313499 10.1126/science.1059495

[169] L. Farnung , S. M. Vos , and P. Cramer , “Structure of Transcribing RNA Polymerase II‐Nucleosome Complex,” Nature Communications 9, no. 1 (2018): 5432. 10.1038/s41467-018-07870-y PMC6303367 30575770

[170] P. J. Robinson , M. J. Trnka , D. A. Bushnell , et al., “Structure of a Complete Mediator‐RNA Polymerase II Pre‐Initiation Complex,” Cell 166, no. 6 (2016): 1411–1422.e16. 27610567 10.1016/j.cell.2016.08.050 PMC5589196

[171] R. B. Berlow , H. J. Dyson , and P. E. Wright , “Hypersensitive Termination of the Hypoxic Response by a Disordered Protein Switch,” Nature 543, no. 7645 (2017): 447–451. 28273070 10.1038/nature21705 PMC5375031

[172] C. N. Johnson , K. A. Sojitra , E. J. Sohn , et al., “Insights Into Molecular Diversity Within the FUS/EWS/TAF15 Protein Family: Unraveling Phase Separation of the N‐Terminal Low‐Complexity Domain From RNA‐Binding Protein EWS,” Journal of the American Chemical Society 146, no. 12 (2024): 8071–8085. 38492239 10.1021/jacs.3c12034 PMC11156192

[173] A. Borgia , M. B. Borgia , K. Bugge , et al., “Extreme Disorder in an Ultrahigh‐Affinity Protein Complex,” Nature 555, no. 7694 (2018): 61–66. 29466338 10.1038/nature25762 PMC6264893

[174] A. Sottini , A. Borgia , M. B. Borgia , et al., “Polyelectrolyte Interactions Enable Rapid Association and Dissociation in High‐Affinity Disordered Protein Complexes,” Nature Communications 11, no. 1 (2020): 5736. 10.1038/s41467-020-18859-x PMC7661507 33184256

[175] P. O. Heidarsson , D. Mercadante , A. Sottini , et al., “Release of Linker Histone From the Nucleosome Driven by Polyelectrolyte Competition With a Disordered Protein,” Nature Chemistry 14, no. 2 (2022): 224–231. 10.1038/s41557-021-00839-3 PMC12810933 34992286

[176] A. Joshi , A. Walimbe , A. Avni , et al., “Single‐Molecule FRET Unmasks Structural Subpopulations and Crucial Molecular Events During FUS Low‐Complexity Domain Phase Separation,” Nature Communications 14, no. 1 (2023): 7331. 10.1038/s41467-023-43225-y PMC10643395 37957147

[177] H. Zhao , J. Li , Y. Xiang , et al., “An IDR‐Dependent Mechanism for Nuclear Receptor Control of Mediator Interaction With RNA Polymerase II,” Molecular Cell 84, no. 14 (2024): 2648–2664.e10. 38955181 10.1016/j.molcel.2024.06.006 PMC11283359

[178] G. A. Armeev , A. S. Kniazeva , G. A. Komarova , M. P. Kirpichnikov , and A. K. Shaytan , “Histone Dynamics Mediate DNA Unwrapping and Sliding in Nucleosomes,” Nature Communications 12, no. 1 (2021): 2387. 10.1038/s41467-021-22636-9 PMC8062685 33888707

[179] E. W. Martin , A. S. Holehouse , I. Peran , et al., “Valence and Patterning of Aromatic Residues Determine the Phase Behavior of Prion‐Like Domains,” Science 367, no. 6478 (2020): 694–699. 32029630 10.1126/science.aaw8653 PMC7297187

[180] A. Bremer , M. Farag , W. M. Borcherds , et al., “Deciphering How Naturally Occurring Sequence Features Impact the Phase Behaviours of Disordered Prion‐Like Domains,” Nature Chemistry 14, no. 2 (2022): 196–207. 10.1038/s41557-021-00840-w PMC8818026 34931046

[181] A. Changiarath , D. Flores‐Solis , J. J. Michels , et al., “Promoter and Gene‐Body RNA‐Polymerase II Co‐Exist in Partial Demixed Condensates,” bioRxiv 2024.03.16.585180; 10.1101/2024.03.16.585180.

[182] G. Fudenberg , M. Imakaev , C. Lu , A. Goloborodko , N. Abdennur , and L. A. Mirny , “Formation of Chromosomal Domains by Loop Extrusion,” Cell Reports 15, no. 9 (2016): 2038–2049. 27210764 10.1016/j.celrep.2016.04.085 PMC4889513

[183] A. L. Sanborn , S. S. P. Rao , S.‐C. Huang , et al., “Chromatin Extrusion Explains Key Features of Loop and Domain Formation in Wild‐Type and Engineered Genomes,” Proceedings of the National Academy of Sciences of the United States of America 112 (2015): E6456–E6465. 26499245 10.1073/pnas.1518552112 PMC4664323

[184] W. Schwarzer , N. Abdennur , A. Goloborodko , et al., “Two Independent Modes of Chromatin Organization Revealed by Cohesin Removal,” Nature 551, no. 7678 (2017): 51–56. 29094699 10.1038/nature24281 PMC5687303

[185] D. T. McSwiggen , M. Mir , X. Darzacq , and R. Tjian , “Evaluating Phase Separation in Live Cells: Diagnosis, Caveats, and Functional Consequences,” Genes & Development 33, no. 23‐24 (2019): 1619–1634. 31594803 10.1101/gad.331520.119 PMC6942051

[186] J. P. Karr , J. J. Ferrie , R. Tjian , and X. Darzacq , “The Transcription Factor Activity Gradient (TAG) Model: Contemplating a Contact‐Independent Mechanism for Enhancer–Promoter Communication,” Genes & Development 36, no. 1‐2 (2022): 7–16. 34969825 10.1101/gad.349160.121 PMC8763055

